# Momentary Mood and Affiliation Following Social Interactions in the Digital Age: Longitudinal Study Investigating Associations With Anxiety and Depression

**DOI:** 10.2196/94753

**Published:** 2026-08-10

**Authors:** Anna Bilston, Sarah Daniels, Yasmin Hasan, Susanne Schweizer

**Affiliations:** 1 School of Psychology, Faculty of Science, UNSW Sydney, Matthews Building, University of New South Wales, Sydney, New South Wales, 2052, Australia, 61 93481896; 2 MRC Cognition and Brain Sciences Unit, University of Cambridge, Cambridge, England, United Kingdom

**Keywords:** adolescence, social media, online social interaction, text messaging, depression, anxiety

## Abstract

**Background:**

Two-fold increases in the prevalence of youth anxiety and depression over the last two decades have mirrored exponential growth in opportunities for adolescent online social interaction via social media, short messaging service (SMS), and internet text messaging apps on smartphones. However, studies to date of self-reported online social interaction time have produced conflicting results. Understanding the role of dispositional and developmental differences in individuals’ responses to online versus offline social interactions may help elucidate whether and how online social interaction is related to anxiety and depression.

**Objective:**

This study aimed to investigate the relationship between older adolescents’ and emerging adults’ (18‐24-year-olds) mental health and (1) objectively measured time spent on smartphones and online social interaction apps, (2) momentary affective and affiliative responses to online and offline social interactions, and (3) the moderating role of developmentally and dispositionally elevated social sensitivity.

**Methods:**

Smartphone, social media (eg, Instagram), SMS, and internet (eg, WhatsApp) text messaging app time from participants’ screen use settings, as well as symptoms of anxiety and depression, and social sensitivity, were measured in 190 older adolescents and emerging adults (mean age 20.4, SD 2.2 years). Participants then completed a novel ecological momentary assessment (EMA) capturing affective and affiliative responses to recent online or offline social interactions 3× daily for 1 week. Symptoms of mental health were assessed again after 1 month.

**Results:**

Total online social interaction (combined social media and text messaging) app time, but not total smartphone time, was associated with greater anxiety, at both baseline and one month later. Affective and affiliative responses were less positive for online social interactions compared to in-person interactions. Anxiety, but not depression, was associated with feeling less happy, but not less included, after social interactions. Affective and affiliative responses to in-person, but not online, social interactions were negatively associated with depression across the 1-month study period. Finally, social sensitivity moderated the relationship between affective and affiliative responses to social media interactions and depression at baseline. Overall effect sizes were small.

**Conclusions:**

These findings emphasize the need to investigate individual factors influencing for whom online social interaction is harmful or beneficial. To do so, this study provides a novel, ecologically valid tool for understanding young people’s momentary responses to online and offline social interactions, as well as initial evidence for stronger associations between in-person than online social interaction responses and mental health for older adolescents and emerging adults. It also introduces evidence of social sensitivity as a potential, developmentally relevant vulnerability to the effects of online social interaction. Further research is needed in younger adolescent populations over longer timeframes.

## Introduction

Human social interaction has been fundamentally transformed in the 21st century by exponential growth in social media and texting apps on smartphones [1]. Traditionally defined, social media refers to internet-based apps enabling user-generated content to be shared from one to many, and texting refers to apps for sending text and images one to one [2-4]; however, the distinction is becoming increasingly less clear [5]. Adolescents and emerging adults (10‐24 years) [6] are frequent users of social media and texting apps, with 96% of 16‐24-year-olds using online social interaction apps for an average of 3 hours per day [1,7]. In parallel to this increased opportunity for online social interaction, we have witnessed annual increases of 12%‐13% in anxiety and depression in 10-19-year-olds globally from 2019 to 2021 [8], reflecting longer-term trends observed in countries across the world, including Australia [9,10] and the United States [11], since the advent of smartphones. Whether these phenomena are causally linked has been the focus of much public and academic debate [12,13]. Efforts to inform the debate should start to unpack the mechanisms through which these apps may be detrimental to mental health.

Prior research has investigated the relationship between adolescent mental health and both device use and social media use (variably defined with and without texting apps included), with a converging view that it is social media use on smartphone devices that is the issue [11]. However, research to date has largely operationalized smartphone or social media use as “time spent,” which has yielded conflicting results [14,15]. Meta-analyses and reviews of reviews have found only a weak positive association [16,17], or no relationship at all [12,18], between the time individuals spent on social media or texting apps and mental health symptoms. One potential reason for these inconsistent results may be the use of memory-reliant, self-reported time spent on smartphones, and social media and texting apps. Several individual [19-22] and meta-analytic studies have demonstrated that self-reported time deviates significantly from logged screentime data [19,23], with one study finding 42% of participants overestimated their time spent online while 26% underestimated their usage [24]. However, Ferguson et al [12] did not find the type of reporting (ie, self-report or objective measurement of time) to be a moderating factor in their meta-analysis of the association between social media and mental health studies. Measurement of time spent may be less accurate when relying on self-report, but it is unlikely to be the sole cause of mixed evidence.

Another source of variation in these associations that is not well captured by time spent on social media is the unique affordances of online interactions. While the concern about online social interactions is that they are detrimental compared with offline (in-person) social interactions [25], few studies have compared young people’s on- and offline interactions. Some of these studies suggest that online sociability has a stronger positive relationship with well-being than offline social activities [26]. However, others indicate that fulfilling self-determination needs (ie, autonomy, relatedness, and competence) through offline, rather than online, connections is predictive of better adolescent mental health [27]. A few studies have used ecological momentary assessments (EMAs) to compare responses to online and offline interactions in real time, or close to real time, in participants’ natural environment [28,29]. This research shows that, on balance, in-person interactions have a more positive effect on mood, feelings of inclusion, and self-esteem than online interactions, both immediately and over time [30-33]. However, these EMA studies did not investigate the link between different momentary responses to online and offline social interactions and mental health.

What’s more, affective and affiliative responses (ie, how positive and included people feel) following different types of social interactions may vary across individuals. The “differential susceptibility to media effects model” [34] posits that responses to media are conditional on interindividual differences in social, developmental, and dispositional susceptibility, and that media effects have a reciprocal causal effect on future media perception and use. Developmentally, online social interaction platforms are particularly attractive to adolescents and emerging adults who are focused on extending their social interactions beyond the family to include large peer networks [35]. However, this increased motivation for peer relatedness also makes adolescence and emerging adulthood a period of greater “social sensitivity” [36] characterized by (1) a focus on social status, approval, and comparison; (2) stronger emotional responses to social evaluation; and (3) more potent effects of negative social experiences on mental health, compared to adults or younger children [37-39]. This arguably makes young people particularly vulnerable to the affordances of online social interaction apps that provide the opportunity for social evaluation and rejection anywhere, anytime, and by anyone [40-42].

Interestingly, when young people were asked about the impact of social media on their well-being, most reported it had no effect and a third reported a positive effect, noting opportunities for social connection [43]. Only 9% reported it had a negative impact on their well-being, although this increased to 32% when asked whether they think social media has a negative impact on *other* “people their age” [43]. Consistent with this, systematic reviews have found that individual responses to online social interactions, such as feeling socially connected or, conversely, let down, are associated with lower anxiety and greater depressive symptoms, respectively [44]. Similarly, while social media use has been cross-sectionally associated with lower well-being and greater levels of anxiety and depression in some samples, changes in social media use were unrelated to changes in mental health within the same individuals over time [45-48]. This suggests that differing mental health outcomes are likely associated with interindividual differences, and maybe not social media itself. Given this, and that adolescents who are high in social sensitivity relative to their peers are more likely to suffer from internalizing disorders [49,50], dispositional as well as developmental social sensitivity represents a potential mechanistic link between the parallel growth in online social interaction and adolescent anxiety and depression [51].

Therefore, the present study aimed to understand whether and how mood and a sense of inclusion (vs exclusion) vary in response to online versus offline interactions and whether they are differentially associated with mental health, specifically symptoms of anxiety and depression. A second aim was to examine whether these associations between affective and affiliative responses to online (vs offline) interactions and mental health varied as a function of individual differences in social sensitivity. These aims were tested in older adolescents and emerging adults aged 18‐24 years, as this age group shows particularly tight coupling between social sensitivity and mental health [52]. Given the focus of the current study on online interactions, we compared responses to in-person interactions to responses to online interactions that captured both social media and texting interactions. Traditionally, there have been significant differences between these modalities, where texting would typically be to one person and social media interactions would include larger audience sizes. However, today social media platforms enable one-to-one communications and texting platforms enable one-to-many communications [2-5], effacing the differences in affordances between the two. Importantly, key affordances of interest including temporal asynchronicity and reduced social cues (compared to in-person interactions) are shared between the modalities. However, to capture any potential differences across modalities, all means of interaction (in-person, social media, texting, and phone/video calls) were captured separately as well.

The current study measured mental health and social sensitivity at the baseline, followed by a high-frequency burst of EMA capturing near real-time responses to online and in-person social interactions, and then mental health was measured again 1 month after baseline. This design allowed us to test the following hypotheses. We hypothesized, first, that participants’ average daily smartphone time, as recorded on their phone, would not be associated with symptoms of anxiety and depression (hypothesis 1a), but that time spent on online social interaction apps would have a small positive association with symptoms of anxiety and depression (hypothesis 1b). Second, we hypothesized that online social interactions would be associated with lower mood and feeling less included compared to in-person social interactions (hypothesis 2). Third, we predicted that greater symptoms of depression and anxiety would be associated with more negative affective and affiliative responses to social interactions (hypothesis 3a), especially for online relative to in-person social interactions (hypothesis 3b). Finally, we hypothesized that the associations between negative responses to online social interaction and poorer mental health (H3) would be potentiated in individuals high in social sensitivity (hypothesis 4).

## Methods

### Participants

Participants were recruited via social media advertising, through an undergraduate psychology course, Prolific, flyers, and word-of-mouth. At baseline, 190 participants (aged 18‐24 years) residing in Australia, the United Kingdom, or the United States were included. About 67% (n=130) of respondents were female, 26% (n=50) were male, and 5% (n=10) had other gender identifications, with moderate ethnic diversity: 52% (n=98) Asian; 28% (n=53) White; 19% (n=37) other ethnicities; and 1% (n=2) preferred not to say. The sample had limited socioeconomic diversity, with 70% (n=133) being highly educated, and 73% (n=140) of respondents perceiving themselves as fairly, rather, or very well-off (Table S1 in Multimedia Appendix 1). Of the 190 participants who completed baseline, 89% (n=169) then completed the week-long EMA and 48% (n=91) completed the 1-month follow-up (Figure S1 in Multimedia Appendix 1). Attrition analyses revealed no significant differences between completers and noncompleters on baseline anxiety, depression, social sensitivity, or screen time variables (Table S2 in Multimedia Appendix 1).

Prolific participants were compensated £9 (GBP £1=US $1.33 as of September 26, 2024) per hour. Undergraduate students were awarded course credits for completion of the baseline assessment and EMA. All other participants were compensated with a Prezee or Amazon voucher in their local currency at US $13.30 per hour for all parts, as were the undergraduate students for the 1-month follow-up. For nonstudent participants, a bonus payment of US $6.50 was awarded if participants completed 80% of the EMA surveys within 1 hour of the notification.

### Measures

#### Mental Health and Individual Differences

##### Anxiety

Symptoms of anxiety were assessed using the 7-item Generalized Anxiety Disorder Scale (GAD-7; see Multimedia Appendix 2) [53]. Frequency of anxiety symptoms, such as “trouble relaxing” in the prior 2 weeks, was rated by participants on a 4-point scale from 0 “not at all” to 3 “nearly every day.” The GAD-7 showed good internal reliability in this sample (ɷ=0.93 baseline, ɷ=0.93 1-month).

##### Depression

Symptoms of depression were measured using the 8-item Patient Health Questionnaire Depression Scale (PHQ-8; see Multimedia Appendix 2) [54]. Frequency of symptoms such as “little interest or pleasure in doing things” in the prior 2 weeks were rated by participants on a 4-point scale from 0 “not at all” to 3 “nearly every-day.” The PHQ-8 showed good internal reliability in this sample (ɷ=0.91 baseline, ɷ=0.90 1-month).

##### Social Sensitivity

Social sensitivity was measured with the 18-item Online and Offline Social Sensitivity Scale (O^2^S^3^; see Multimedia Appendix 2) [52]. The O^2^S^3^ assesses social rejection sensitivity across offline and online social interactions. Participants indicated the extent to which they agreed with statements such as “I worry about being criticized for things I have said or done” (worry about approval); “If others knew the real me, they would not like me” (social investment); “I feel anxious before posting anything on social media” (social risk); and “I delete my social media posts if I don’t get the responses I wanted” (online social interaction). All responses were answered on a 4-point Likert scale ranging from 0 (“strongly disagree”) to 3 (“strongly agree”). The O^2^S^3^ demonstrated good reliability in this sample (ɷ=0.92 baseline, ɷ=0.94 1-month).

##### Time Spent

As an uncomplicated, user-centric method for data collection [55,56], and consistent with similar recent studies [57], time spent on smartphones, social media, and texting time was obtained from smartphone-logged data provided by participants as screenshots. All screenshots were reviewed, and data were inputted manually to ensure validity and completeness. Time spent on smartphones was determined using the average daily screentime calculated from participants’ screenshots at baseline and after the 1-week EMA to create a single representative average time. Social media and texting time was calculated as the sum of the time participants spent on social media and texting apps on their smartphones across the week, as provided in participant screenshots, converted to a daily average time. Online social interaction time is the average daily social media and texting time combined. See Table S3 in Multimedia Appendix 1 for a list of online social interaction apps included.

##### Timepoint

Defined as timepoint 1 (baseline) and timepoint 2 (1-month follow-up).

### Ecological Momentary Assessment

#### Frequency and Duration

Participants were sent EMA surveys 3 times a day for 7 days, resulting in 21 EMA surveys [58,59]. To optimize ecological validity, each prompt was scheduled for different times within a period from 45 minutes to 1 hour 45 minutes each day, and therefore appeared random to participants. Prompt 1 was sent between 10 AM and 11:45 AM, prompt 2 was sent between 3 PM and 4 PM, and prompt 3 was sent between 8 PM and 8:45 PM, with an average time of 4.5‐5 hours between prompts. Participants were required to respond to each prompt within 2 hours of it being sent.

#### Items

For each survey (Multimedia Appendix 2), participants answered multiple-choice questions about the “single most important connection” [33] experienced in the “past 2 hours” [60]. Based on connection types assessed in prior EMA studies [33,61,62], participants identified if the connection was “in-person/real-life,” “phone/voice/video call,” “text message/instant message/SMS/WhatsApp/email,” “social media (eg, Instagram, TikTok, Snapchat, and LinkedIn),” or “other” [62]. The current study investigated in-person versus online interactions, with online interactions operationalized as social media and digital messaging: a composite of “social media (eg, Instagram, TikTok, Snapchat, and LinkedIn)” and “text message/instant message/SMS/WhatsApp/email.” However, for completeness, analyses separating each connection type (ie, in-person, social media, messaging, and phone) are reported in Multimedia Appendix 1, and notable deviations for texting or social media are reported in the main manuscript.

Responses to social interactions were assessed with the EMA question “how did the social interaction make you feel overall?” Two visual analog scales from 0 to 100 measured affective response, operationalized as happiness (from “very unhappy” to “very happy”) [63], and affiliative response, operationalized as feeling included (from “left out” to “included”) [64]. To ensure each survey took approximately the same time to complete regardless of whether participants had interacted socially or not [65], if participants had not had a social interaction in the prior 2 hours, they were asked questions about why they had not, how usual this was, and whether they viewed this time not socializing as positive or negative.

### Ethical Considerations

Ethics approval for this study was granted by the UNSW Human Research Ethics Advisory Panel (HREAP6389).

Participants were provided with comprehensive participant information, including the opportunity to opt out for any reason at any time, and provided consent online via Qualtrics before responding to part 1 questionnaires. Participant privacy was managed according to the UNSW Privacy Management Plan, a copy of which was made accessible to participants. Before analysis, all identifying data were removed and replaced with a randomly generated code. Identifying information was kept separately for the sole purpose of reidentifying participants wishing to withdraw from the study.

### Procedure

After providing informed consent, participants completed the baseline survey measuring demographic characteristics, along with mental health symptoms and individual differences, followed by 7 days of EMA surveys. EMA survey links were sent to Prolific participants via email, and all other participants received prompts via SMS. The baseline survey (with the exception of demographics) was completed again after 1 month. At the end of the baseline assessment and the EMA survey period, participants also uploaded screenshots of the total average daily time spent on their device, as well as weekly time spent on each app. All surveys and uploads were completed in Qualtrics.

### Data Analysis

All analyses were conducted in R (version 4.4.2).

All linear mixed models included random intercepts for participant ID; these are not noted again in this section. Standardized predictors were used in all regression-based models. Outlier analysis using the IQR method (1.5 × IQR below Q1 or above Q3) indicated a small number of outliers in responses by connection type (range: 0‐3 per group), consistent with natural variation in a community sample. Outliers were therefore not removed. Repeating primary analyses excluding these outliers did not change the pattern of results.

Total participant device (H1a) and online social interaction time (H1b) were included as fixed factors in separate linear regression models, with baseline anxiety and depression scores as outcomes. These relationships were also investigated longitudinally across the 1-month follow-up period using linear mixed models with timepoint as within-subjects fixed effects and time spent on devices and online social interaction as between-subjects fixed effects.

Mixed models including EMA timepoint (1-21) were all run twice: once with EMA timepoint as an independent within-subjects fixed effect and once with EMA timepoint allowed to interact with the other fixed effects in the model. Model fit indices were then compared, and if formal comparison yielded a significant difference between models, the better-fitting (lower Akaike information criterion by at least two points) model was chosen. If the model fit was not significantly different between models, the more parsimonious (without interaction term) model was selected.

To test H2, EMA timepoint and connection type (in-person vs online) were included as within-subject effects. To examine the association between anxiety and depression symptoms and responses to social interaction (H3a), linear mixed models including EMA timepoint as within-subjects effects and symptoms of anxiety and depression as between-subjects effects were built. Connection type was then added to the H3a model as a within-subjects fixed effect to test whether the effect of mental health symptoms differed between online and in-person social interactions (H3b). Intraclass correlation coefficients (ICCs) for these models ranged from 0.26 to 0.41 across anxiety and depression outcomes, indicating a high level of within-person variability and within accepted thresholds for mixed modeling [66,67]. To examine these associations (H3) over time, mean affective and affiliative response scores collected across the 7-days of EMA surveys were calculated for each participant, scaled, and then included as a fixed between-subjects effect in a model including timepoint (baseline vs 1-month) as a fixed within-subjects effect to predict symptoms of anxiety. ICCs for these longitudinal models ranged from 0.62 to 0.67 across anxiety and depression [66,67]. Finally, to assess whether the associations between mean responses to social interactions and mental health outcomes were moderated by baseline social sensitivity (H3), both cross-sectionally (between participants at each timepoint) and longitudinally (within participants between timepoint 1 and timepoint 2), linear regression and linear mixed models were used, respectively.

Effect sizes for all models were reported as partial eta-squared (η^2^_p_), which represents the proportion of variance explained by the relevant variable, and is therefore equal to eta-squared for linear regression models. Eta-squared of .01, .06, and .14 are considered small, medium, and large effect sizes, respectively [68]. All significant interaction effects were examined with simple slope analyses.

For all EMA data analyses (H2, H3a/b), restricted maximum likelihood (REML) estimation was used [69], thereby using all available social interaction responses contributed by each participant, regardless of how many social interactions they reported. This approach provides unbiased parameter estimates under the assumption that data are missing at random [70]. Responses from prompts where no social interaction was reported were not included in the present analyses. The analyses including baseline and 1-month as timepoints applied listwise deletion, thereby only including participants for whom there was data at both timepoints to ensure meaningful analysis.

Usage time predictors (device time, social media time, texting time, and combined social interaction time) were specified prior to analysis to enable direct comparison with previous literature that has differentially operationalized screen and social media time exposure. Moderation analyses were similarly theory-driven, based on a priori hypotheses derived from the “differential susceptibility to media effects” model and prior research. No additional correction for these predictors was therefore applied. For the two outcomes of interest (anxiety and depression symptoms), the α threshold was set to .025, applying a Bonferroni correction such that an overall family-wise error rate of .05 was maintained across both outcomes, with tests set as 2-tailed. Post hoc analyses were completed via pairwise comparisons with Tukey adjustment to maintain an overall α threshold of .05.

Sensitivity analyses were conducted for all primary analyses, restricting the sample to participants who completed at least 50% of EMA surveys (≥7 of 21 surveys; 112 participants). Effects were consistent in direction and significance across the full and 50% minimum completion samples, indicating the moderate compliance rate did not systematically bias the results (Tables S4-S6 in Multimedia Appendix 1).

## Results

### Compliance and Reactivity

Overall, 169 participants completed at least 1 EMA, resulting in 2306 EMA survey responses. Of the 21 EMA surveys, participants completed 13.6 (SD 6.9) on average. Average compliance, therefore, was 65% (SD 33%; range 5%‐100%), with a good average response time (within 37.5 minutes of the prompt). Neither symptoms of anxiety (β=0, *t*_167_=0.03, *P*=.98) nor depression (β=0, *t*_167_=0.36, *P*=.72) were significantly associated with EMA compliance rate. Attrition analyses comparing completers and noncompleters of the 1-month follow-up found that completers demonstrated significantly higher EMA compliance than noncompleters (76% vs 54%; *t*_151_=−4.69, *P*<.001; Table S2 in Multimedia Appendix 1). Reactivity was minimal with no significant change in participant time spent on either their smartphone (*t*_94_=−0.39, 95% CI −27.51 to 18.48; *P*=.70) or online social interaction (*t*_73_=−1.04, 95% CI −26.79 to 8.40; *P*=.30) from baseline to after the EMA.

### Association Between Time Spent on Smartphones and Online Social Interaction Applications and Mental Health

Participants spent an average of 6 hours and 23 minutes per day on their phones (SD*=*3 hours and 11 minutes), of which 2 hours and 32 minutes (SD=1 hour and 49 minutes) was spent using online social interaction apps. There was no significant association between time spent on smartphones and symptoms of anxiety and depression at baseline (Table 1) or at the 1-month follow-up (Table 2). However, at baseline, average daily time spent on online social interaction apps was associated with both higher anxiety and depressive symptoms (Table 1).

**Table 1.** Relationship between average daily smartphone and online social interaction time and mental health at baseline.

	Anxiety^a^					Depression^b^				
Predictor	β	SE	*t* test^c^ (*df*)	*P* value^d^	η^2^_p_^e^	β	SE	*t* test^c^(*df*)	*P* value^d^	η^2^_p_^e^
Smartphone time^f^	−0.04	0.08	−0.49	.63	.00	−0.03	0.08	−0.42	.67	.00
Online social interaction time^f^	0.29	0.08	3.70	*<.001*	.09	0.19	0.08	2.31	*.02*	.04

a Total score on the 7-item Generalized Anxiety Disorder Scale (GAD-7) [53].

b Total score on the 8-item Patient Health Questionnaire (PHQ-8) [54].

c Two-tailed *t* test with α threshold of .025.

d Significant results at an α threshold of .025 are italicized.

e η2_p_ values of .01, .06, and .14 are considered small, medium, and large effect sizes, respectively [68].

f Smartphone and online social interaction time are operationalized as average daily minutes spent on smartphones and the combined time spent on social media and texting apps, respectively.

Across the 1-month follow-up period, there were no significant changes in anxiety (*F*_1,104_=2.06, *P=*.15, η^2^_p_=.02) or depression (*F*_1,109_=0.29, *P=*.59, η^2^_p_=.00). The small association between online social interaction time and anxiety remained significant; however, there were no significant associations between online social interaction time and symptoms of depression across the 1-month follow-up period (Table 2). There were no notable differences in the relationship between social media time and texting time, and mental health symptoms (Tables S7 and S8 in Multimedia Appendix 1).

**Table 2.** Relationship between average daily smartphone and online social interaction time and mental health over time.

		Anxiety^a^			Depression^b^		
Predictor		*F* test *(df)*	*P* value^c^	η^2^_p_^d^	*F* test (*df)*	*P* value^c^	η^2^_p_^d^
Smartphone time							
Timepoint^e^		3.02 (1, 98)	.09	.03	0.00 (1, 101)	.95	.00
Smartphone time^f^		0.11 (1, 174)	.74	.00	0.03 (1, 175)	.87	.00
Smartphone time × timepoint		0.12 (1, 96)	.73	.00	0.25 (1, 99)	.62	.00
Online social interaction (OSI) time							
Timepoint^e^		2.35 (1, 82)	.13	.03	0.07 (1, 79)	.80	.00
OSI time^f^		7.07 (1, 188)	*.01*	.04	3.09 (1, 187)	.08	.02
OSI time × timepoint		1.72 (1, 87)	.19	.02	0.49 (1, 84)	.49	.01

a Total score on the 7-item Generalized Anxiety Disorder Scale (GAD-7) [53].

b Total score on the 8-item Patient Health Questionnaire (PHQ-8) [54].

c Significant results at an α threshold of .025 are italicized.

d η2_p_ values of .01, .06, and .14 are considered small, medium, and large effect sizes, respectively [68].

e Timepoint*=*baseline vs 1-month follow-up.

f Smartphone and online social interaction time are operationalized as average daily minutes spent on smartphones and the combined time spent on social media and texting apps, respectively.

### Responses to Online and Offline Social Interactions, and the Association With Mental Health

Of the 1598 EMAs where participants had interacted socially in the prior 2 hours, 67% (n=1074) of their most important social interactions were in-person, while 20% (n=323) of their most important interactions were online via social media or texting apps (Table S9 in Multimedia Appendix 1).

For all analyses including the EMA timepoint, the model adding the EMA timepoint as a fixed effect that did not interact with other within- and between-subjects effects showed the best fit (Table 3).

**Table 3.** Comparison of mixed models with ecological momentary assessment (EMA) as fixed effect versus moderator.

	Affective^a^				Affiliative^a^			
Hypothesis: model predictor	AIC^b^ (model + EMA^c^)	AIC (model * EMA^c^)	Chi-square (*df*)	*P* value^d^	AIC (model + EMA^c^)	AIC (model * EMA^c^)	Chi-square (*df*)	*P* value^d^
H2: connection type^e^	12,059	12,070	28.96 (20)	.09	11,925	11,940	25.24 (20)	.19
H3a: mental health^f^								
Anxiety	12,083	12,093	30.01 (20)	.07	11,946	11,953	32.55 (20)	.04
Depression	12,084	12,096	27.43 (20)	.12	11,946	11,957	29.41 (20)	.08
H3b: connection typemoderated by mental health								
Anxiety	12,056	12,086	90.44 (60)	*.01*	11,926	11,963	82.31 (60)	.03
Depression	12,057	12,091	85.91 (60)	*.02*	11,927	11,967	79.61 (60)	.05

a At each EMA, participants reported their affective and affiliative response to their most important social interaction in the past 2 hours on scales ranging from “0=very unhappy” to “100=very happy” and from “0=left out” to “100=included,” respectively.

b AIC: Akaike information criterion.

c 21 EMA timepoints: 3/day for 7 days.

d Significant results at an α threshold of .025 are italicized.

e In-person and via online social interaction (social media and texting) apps.

f Anxiety=total score on the 7-item Generalized Anxiety Disorder Scale (GAD-7) [53]. Depression=total score on the 8-item Patient Health Questionnaire (PHQ-8) [54].

**Figure 1.**
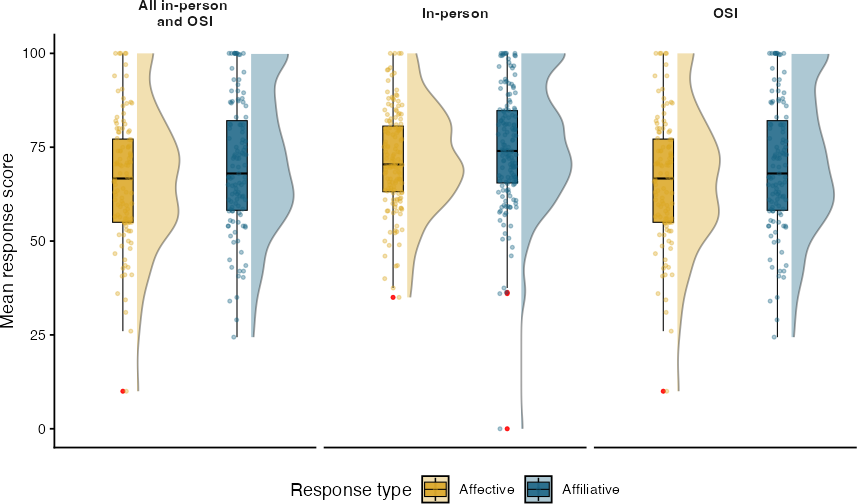
Affective and affiliative responses to in-person and online social interactions (OSI): individual distributions and median and IQR for means across all ecological momentary assessments.

The model H2 showed no main effect of EMA timepoint (affective: *F*_20,1265_=1.48, *P=*.08, η^2^_p_=.02; affiliative: *F*_20,1251_=1.54, *P=*.06, η^2^_p_=.02) but affective (*F*_1,1353_*=*29.01*, P*<.001, η^2^_p_=.02) and affiliative (*F*_1,1321_*=*23.35*, P*<.001, η^2^_p_=.02) responses varied by connection type (Figure 1). Specifically, affective responses to online social interactions were consistently less positive than responses to in-person social interactions (β=−6.51, 95% CI −4.14 to −8.89). The same pattern emerged for affiliative responses (β*=*–5.51, 95% CI −3.27 to −7.75), with individuals feeling less included following online relative to in-person interactions across EMA timepoints. The direction of the relationship between responses and both social media and texting was consistent (Table S10 in Multimedia Appendix 1).

Models H3a predicting affective responses to social interactions showed a significant main effect of symptoms of anxiety (*F*_1,151_=5.42, *P*=.02, η^2^_p_=.03) but not depression (*F*_1,151_=4.71, *P*=.03, η^2^_p_=.03). Greater symptoms of anxiety (β=−2.33, 95% CI −4.29 to −0.37) were associated with more negative affective responses to social interactions. Adding connection type to the model showed that the main effect of connection type remained significant when accounting for mental health, but mental health symptoms did not interact with connection type (Table 4). That is, affective responses to online social interactions were less positive compared to responses to in-person interactions irrespective of individuals’ symptoms of anxiety or depression (Table S11 in Multimedia Appendix 1). Baseline mental health symptoms were not associated with how included individuals felt in response to social interactions (anxiety: *F*_1,157_=3.05, *P*=.08, η^2^_p_=.02; depression: *F*_1,157_=2.25, *P*=.14, η^2^_p_=.01). Adding connection type to the model showed no significant interaction between mental health symptoms and connection type (Table 4).

**Table 4.** Relationship between connection type and momentary affective and affiliative responses, moderated by baseline anxiety and depression.

		Affective response^a^			Affiliative response^a^		
		*F* test (*df*)	*P* value^b^	η^2^_p_^c^	*F* test (*df*)	*P* value^b^	η^2^_p_^c^
Anxiety							
EMA^d^		1.47 (20, 1264)	.08	.02	1.55 (20, 1250)	.06	.02
Anxiety^e^		3.77 (1, 190)	.05	.02	2.65 (1, 184)	.11	.01
Connection type^f^		28.49 (1, 1356)	*<.001*	.02	23.11 (1, 1324)	*<.001*	.02
Anxiety × connection type		0.90 (1, 1367)	.34	.00	0.18 (1, 1343)	.67	.00
Depression							
EMA^d^		1.45 (20, 1263)	.09	.02	1.54 (20, 1250)	.06	.02
Depression^e^		3.01 (1, 181)	.08	.02	1.95 (1, 178)	.16	.01
Connection type^f^		28.32 (1, 1356)	*<.001*	.02	23.08 (1, 1324)	*<.001*	.02
Depression × connection type		1.34 (1, 1367)	.25	.00	0.11 (1, 1353)	.74	.00

a At each ecological momentary assessment (EMA), participants reported their affective and affiliative response to their most important social interaction in the past 2 hours on scales ranging from “0=very unhappy” to “100=very happy” and from “0=left out” to “100=included,” respectively.

b Significant results at an α threshold of .025 are italicized.

c η2_p_ values of .01, .06, and .14 are considered small, medium, and large effect sizes, respectively [68].

d 21 EMA timepoints: 3/day for 7 days.

e Anxiety=total score on the 7-item Generalized Anxiety Disorder Scale (GAD-7) [53]. Depression=total score on the 8-item Patient Health Questionnaire (PHQ-8) [54].

f In-person and via online social interaction (social media and texting) apps.

Across time, from baseline to 1-month, average affective and affiliative responses to in-person social interactions were inversely associated with depressive symptoms (Table 5), with the effect remaining stable across time (ie, no response × time interaction). Online social interactions were not significantly associated with mental health symptoms across 1-month (Table 5). This was consistent for both social media and texting interactions (Table S12 in Multimedia Appendix 1)

**Table 5.** Relationship between affective and affiliative responses to social interactions and mental health over time (baseline and 1-month), for in-person interactions and online social interactions.

		Anxiety^a^			Depression^b^		
		*F* test (*df*)	*P* value^c^	η^2^_p_^d^	*F* test (*df*)	*P* value^c^	η^2^_p_^d^
In-person interactions							
Affective response							
Timepoint^e^		2.29 (1, 92)	.13	.02	0.08 (1, 93)	.77	.00
Affective response^f^		4.63 (1, 158)	.03	.03	7.39 (1, 157)	*.01*	.04
Affective response × timepoint		0.03 (1, 92)	.86	.00	1.68 (1, 93)	.20	.02
Affiliative response							
Timepoint^e^		2.26 (1, 92)	.14	.02	0.22 (1, 93)	.64	.00
Affiliative response^f^		2.76 (1, 158)	.10	.02	7.42 (1, 158)	*.01*	.04
Affiliative response × timepoint		0.01 (1, 92)	.92	.00	2.85 (1, 93)	.10	.03
Online social interactions							
Affective response							
Timepoint^e^		0.47 (1, 75)	.50	.01	0.04 (1, 79)	.85	.00
Affective response^f^		0.53 (1, 123)	.47	.00	0.03 (1, 123	.87	.00
Affective response × timepoint		0.87 (1, 75)	.35	.01	0.16 (1, 79)	.69	.00
Affiliative response							
Timepoint^e^		0.36 (1, 76)	.55	.00	0.16 (1, 79)	.69	.00
Affiliative response^f^		1.23 (1, 122)	.27	.01	3.20 (1, 122)	.08	.03
Affiliative response × timepoint		0.25 (1, 75)	.62	.00	1.83 (1, 78)	.18	.02

a Total score on the 7-item Generalized Anxiety Disorder Scale (GAD-7) [53].

b Total score on the 8-item Patient Health Questionnaire (PHQ-8) [54].

c Significant results at an α threshold of .025 are italicized.

d η2_p_ values of .01, .06, and .14 are considered small, medium, and large effect sizes, respectively [68].

e Timepoint*=*baseline vs 1-month follow-up.

f At each EMA, participants reported their affective and affiliative response to their most important social interaction in the past 2 hours on scales ranging from “0=very unhappy” to “100=very happy” and from “0=left out” to “100=included,” respectively.

### Moderating Role of Social Sensitivity on the Association Between Affective and Affiliative Responses to Social Interactions and Mental Health

**Figure 2.**
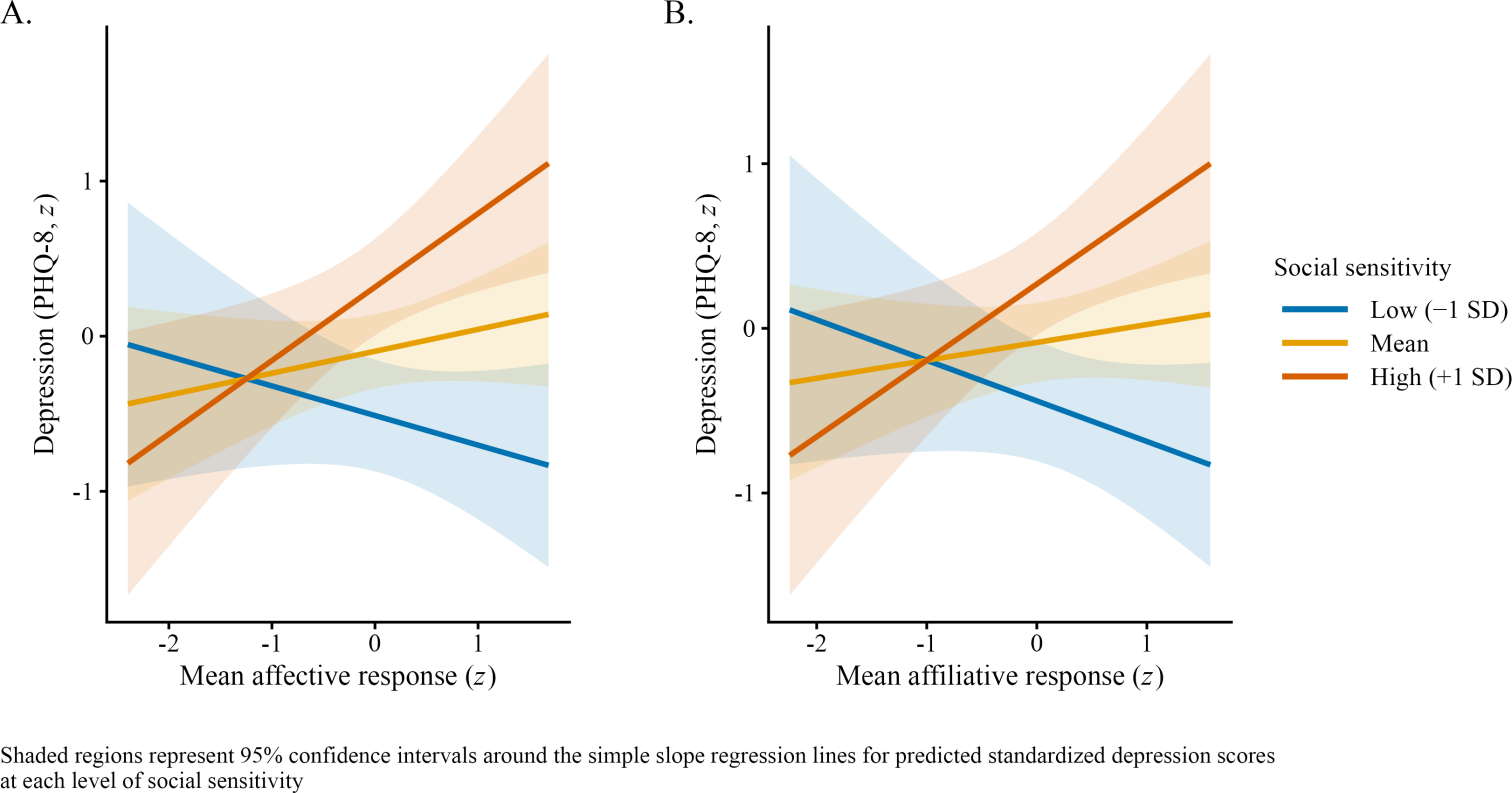
Relationship between (A) affective and (B) affiliative responses to social media interactions and depression, moderated by social sensitivity at baseline. PHQ-8: 8-Item Patient Health Questionnaire Depression Scale.

While higher social sensitivity, as measured at baseline, was associated with higher levels of anxiety and depression at both baseline and 1-month follow-up, social sensitivity did not moderate the relationship between individuals’ average affective or affiliative responses to online or in-person social interactions and mental health, at either timepoint (Tables S13 and S14 in Multimedia Appendix 1). However, social media interactions (without texting) showed a different pattern of association, and as they deviate from the “online interaction findings,” they are reported in the main manuscript. Social sensitivity moderated the association between baseline depressive symptoms and both affective (*F*_1,39_=6.84, *P=*.01, η^2^_p_=.15) and affiliative (*F*_1, 39_=6.77, *P=*.01, η^2^_p_=.15) responses to social media interactions (Table 6). Post hoc simple slopes analyses indicated that this relationship was only significant for individuals with relatively high levels of social sensitivity (+1SD), such that more positive affective (β*=*2.65, *t*_39_=2.66, *P=*.01, 95% CI 0.63-4.66) and affiliative (β*=*2.59, *t*_39_=2.52, *P=*.02, 95% CI 0.51-4.60) responses to social media interactions were associated with greater depressive symptoms (Figure 2). However, this interaction varied by timepoint, such that the significant moderating effect of social sensitivity on the association between affective and affiliative responses and depression was not maintained at the 1-month follow-up (Table S14 in Multimedia Appendix 1).

**Table 6.** Baseline association between affective and affiliative responses and anxiety and depression, moderated by social sensitivity for social media interactions only.

	Anxiety^a^			Depression^b^		
Predictor	*F* test (*df*)	*P* value^c^	η^2^_p_^d^	*F* test (*df*)	*P* value^c^	η^2^_p_^d^
Affective response	(1, 39)			(1, 39)		
Affective response^e^	0.40	.53	.01	0.56	.46	.01
Social sensitivity^f^	9.74	*.003*	.20	10.41	*.003*	.21
Affective response × social sensitivity	0.40	.53	.01	6.84	*.01*	.15
Affiliative response	(1, 39)			(1, 39)		
Affiliative response^e^	1.18	.28	.03	0.28	.60	.01
Social sensitivity^f^	9.85	*.003*	.20	10.14	*.003*	.21
Affiliative response × social sensitivity	1.61	.21	.04	6.77	*.01*	.15

a Anxiety=total score on the 7-item Generalized Anxiety Disorder Scale (GAD-7) [53].

b Depression=total score on the 8-item Patient Health Questionnaire (PHQ-8) [54].

c Significant results at an α threshold of .025 are italicized.

d η2_p_ values of .01, .06, and .14 are considered small, medium, and large effect sizes, respectively [68].

e At each EMA, participants reported their affective and affiliative response to their most important social interaction in the past 2 hours on scales ranging from “0=very unhappy” to “100=very happy” and from “0=left out” to “100=included,” respectively.

f Social sensitivity=total score on the 18-item Online and Offline Social Sensitivity Scale (O2S3) [52].

## Discussion

### Principal Results

Rising rates of youth anxiety and depression have coincided with increased smartphone use and opportunities for online social interaction, fueling a popular belief that online social interaction is inherently harmful. However, meta-analyses have yielded mixed findings [12], highlighting the need for identifying individual differences in susceptibility to the effects of online social interaction. As hypothesized, and consistent with the existing literature [16,17,71,72], there was no significant relationship between symptoms of anxiety and depression and objectively measured smartphone time, and only small associations with online social interaction time. However, participants felt less positive and included after online social interactions than in-person interactions. Interestingly, baseline anxiety symptoms were negatively associated with how happy someone felt after social interactions, but there was no significant relationship with depression, nor between feelings of inclusion and anxiety or depression. Further, over the month of the study, while feeling more positive and included after in-person social interactions was associated with lower depression symptoms, responses to online social interactions were not associated with mental health, and these associations were not moderated by social sensitivity. However, social media differed from the overall online interactions here. Notably, individuals higher in social sensitivity showed a positive association between depressive symptoms at baseline and more positive and stronger affiliative responses to online social interactions. This moderating effect of social sensitivity was not maintained at 1-month. Together, these findings highlight the need for elucidating dispositional and developmental sensitivity to online interactions to develop a more nuanced understanding of their associations with mental health.

Interestingly, given reports of many 13‐19-year-old adolescents using social media “almost constantly” [71,72], across the week of EMA, these 18‐24-year-old participants disproportionately chose in-person interactions as their “most important” recent interaction. Consistent with previous EMA studies [30,32,73], participants reported feeling more positive and included during in-person, compared to online, social interactions. Feeling less positive and less included after online social interactions compared to in-person interactions may have been due to temporal asynchrony (eg, delayed replies) and impoverished social cues that lead to a perception of reduced thoughtfulness and empathy [25,42,74,75]. Alternatively, online social interactions may elicit less positive feelings than in-person interactions because they can be used for less emotionally charged purposes, such as exchanging information or arranging meetings [76] via text. Richer contextual cues, as well as differing affordances and motivations for in-person compared to online interactions, may therefore make offline connections more meaningful. Future research would benefit from directly comparing the mechanisms through which each modality affects mood and feelings of inclusion. For example, examining whether synchronicity, audience closeness and size, or social evaluation features differentially predict affective and affiliative responses to online interactions across modalities.

The finding that anxiety symptoms were associated with participants feeling less happy after social interactions is consistent with the high correlation between adolescent anxiety and negative interpretation bias in social situations [49], which has been found to increase with adolescent age [77]. It is also consistent with recent meta-analyses of prospective studies, which found that anxiety in adolescents across the age spectrum cultivates lower levels of companionship, intimacy, and support in relationships [78,79], arguably the factors that make social interactions a positive experience. The lack of association with symptoms of depression may be a power issue, with the effect size and direction of the association being the same as for anxiety.

In contrast, the positive association between mood and feelings of inclusion following in-person social interactions and mental health is consistent with evidence that emerging adults’ ability to meet their needs for autonomy, relatedness, and competence is optimized by engaging in mostly offline social interaction [80]. While previous studies have found there can be enhancement effects when online social interaction complements offline interaction [25], these findings suggest that if online social interactions displace in-person social interactions, young people may feel relatively less happy and included more often. Future research is needed to examine whether online interactions are indeed displacing in-person interactions. The introduction of the social media age restrictions in Australia offers an interesting opportunity to test the displacement hypothesis by comparing online versus offline social interaction frequency in adolescents in a country with government-restricted access, to online versus offline interaction ratios in adolescents from other countries.

In line with the drive toward identifying individual differences that account for the mental health impacts of online interactions, this study examined the effects of social sensitivity, which was associated with anxiety and depressive symptoms. Counterintuitively, individuals high in social sensitivity and depressive symptoms responded more positively to social media interactions. Importantly, given the cross-sectional nature of this association, the risk of reverse causality cannot be eliminated, and several interpretations of this finding warrant consideration. First, more positive responses to online social interactions for those high in social sensitivity may reflect social avoidance, where participants who are especially high in social sensitivity are more likely to avoid in-person interactions during which they fear getting rejected [81]. This is consistent with compensatory online use, where socially anxious individuals perceive online interactions more positively, as a format offering greater control [82]. Alternatively, this finding is also consistent with the social risk hypothesis [81], where people with mild to moderate depression are motivated by a fear of social rejection to seek safe forms of social contact in order to restore their social value. Social media may offer a means to carefully curate an interaction to minimize the risk of social rejection, thus resulting in greater momentary positive affect and feeling included. However, in the longer term, these interactions may be unfulfilling [83], drive lower feelings of self-worth via pressure to maintain social relevance [84], and/or promote negative self-comparisons [85]. Equally, it may be that those who are socially sensitive and experiencing anhedonia due to depression find the energy requirements of in-person social interactions excessive, but are able to selectively engage with, and respond more positively to, online social interactions.

In contrast with this argument is the finding that the moderating effect of social sensitivity was not maintained at the 1-month follow-up. The positive affect experienced during online interactions may have offered support for their mental health challenges over the month-long period [86,87], though this hypothesis will need to be explicitly tested through future research.

Additionally, effect sizes for all observed relationships in this study were small, emphasizing the multifactorial nature of influences on adolescent mental health and highlighting that understanding individual differences in susceptibility to the pros and cons of online social interactions is a promising avenue for studying its mental health impacts. Further, small effects may still have meaningful implications when considered at the population level, especially among younger adolescents who are arguably more vulnerable to the effects of social media [88], and given the ubiquity of social media use by young people. Therefore, even modest effects may indicate opportunities for intervention.

### Strengths and Limitations

These results should be considered within the context of the study’s strengths and limitations. The study’s main strengths were (1) high-frequency assessment in the real-world, minimizing recall bias [30] (ie, remembering the prior 2 hours vs 2 weeks); (2) testing of theory-driven individual differences in susceptibility to social media effects; and (3) using an objective assessment of time spent on smartphones and social media apps (screenshot). However, the observational study design precludes causal inferences about the role of online versus in-person social interaction on mental health.

The study findings should be interpreted in the context of our specific sample of older adolescents and emerging adults aged 18‐24 years, who volunteered to participate, and 73% (n=140) of whom described themselves as fairly to very well-off. This means these results may not generalize to younger, less wealthy adolescents, or those unlikely to volunteer, for whom responses, mental health, and developmental differences in emotional regulation and social sensitivity may differ. Equally, it is possible that those who completed all parts of the study were more motivated, conscientious, or digitally engaged than noncompleters, creating systematic bias toward those with these personality traits and interests [89]. Taken together, this limits the generalizability of findings. Further, while 98% of social media users connect via smartphones [90], online social interaction time may still have been underestimated for some participants by only capturing time on smartphones, as opposed to other digital devices. This is especially the case for Android users, for whom detailed app usage data was less available. Further, using participant-uploaded screenshots may have limited data collection as inaccurate screenshots could not be used, and in the era of AI, screenshots could have been subject to manipulation. However, this is unlikely as there was no incentive for individuals to alter screenshots provided, and there was a disincentive given it would be time-consuming to do so. Smartphone sensing and digital phenotyping are more reliable means to capture time spent online in future studies.

While the novel EMA used in this study enabled a unique view of adolescents’ social experiences while minimizing recall bias [30], EMA compliance of 65% was less than the 79% average found in a recent meta-analysis of EMA studies [59]. However, overall attrition at follow-up was also substantial (48%), and once noncompleters were removed, EMA compliance was 76%, with sensitivity analyses indicating no change in the direction or significance of results. Additionally, EMA compliance rates have been found to be substantially higher in older, compared to younger, adults [91], and therefore, there may not have been as much of a difference in compliance in this study compared with other studies also exclusively involving emerging adults. Regardless, identifying ways to increase young people’s compliance would optimize the analytical power and efficiency of future studies.

Finally, while the 1-month follow-up period was sufficient to examine short-term longitudinal associations, future research should use extended follow-up periods to enable the detection of longer-term effects of online social interaction on mental health trajectories.

### Conclusions

Overall, this study supports the literature to date indicating that time spent on smartphones and online social interaction is only weakly correlated with older adolescent mental health. It also extends evidence of online social interactions as mostly positive experiences to older adolescents and emerging adults; albeit, these experiences may not make adolescents feel as happy or as included relative to in-person interactions. To better understand what makes online social interaction harmful for some young people and not others, this study offers an EMA blueprint for evaluating adolescents’ momentary experiences of online and offline social interactions, as well as individual and developmental susceptibilities to online social interaction effects. It also provides preliminary evidence of the potential moderating role of social sensitivity on the relationship between adolescents’ responses to social interactions and mental health outcomes. Further research across the adolescent age spectrum is needed to understand the individual characteristics that influence adolescents’ vulnerability to both positive and negative online versus offline social interactions.

## Supplementary material

10.2196/94753 Multimedia Appendix 1 Participant demographics and sensitivity and other additional analyses.

10.2196/94753 Multimedia Appendix 2 Survey questionnaires.

## Abbreviations

EMA: ecological momentary assessment
GAD-7: 7-Item Generalized Anxiety Disorder Scale
ICC: intraclass correlation coefficient
O^2^S^3^: 18-Item Online and Offline Social Sensitivity Scale
PHQ-8: 8-Item Patient Health Questionnaire Depression Scale
REML: restricted maximum likelihood
