# Supplementary material for: Momentary Mood and Affiliation Following Social Interactions in the Digital Age: Longitudinal Study Investigating Associations With Anxiety and Depression
Source: JMIR Ment Health. 2026 Aug 10;13:e94753. doi: 10.2196/94753 (PMC13455724; doi:10.2196/94753)
Supplement: Supplementary file 1 [file mental-v13-e94753-s001.docx]

Multimedia Appendix 1: Participant demographics and sensitivity and other additional analyses

Table S1. Participant demographic characteristics.

| Variable | | Part 1  *(N*=190) | Part 2  (*N*=169) | Part 3  (*N*=91) |
| --- | --- | --- | --- | --- |
| Age in years, M (SD) | | 20.4 (2.2) | 20.5 (2.2) | 20.5 (2.2) |
| **Gender, n (%)** | |  |  |  |
|  | Female | 130 (68) | 116 (69) | 66 (73) |
|  | Male | 50 (26) | 44 (26) | 22 (24) |
|  | Non-binary / Third gender / Other | 10 (5) | 9 (5) | 3 (3) |
| **Country, n (%)** | |  |  |  |
|  | Australia | 139 (73) | 127 (75) | 63 (69) |
|  | United States of America | 35 (18) | 29 (17) | 18 (20) |
|  | United Kingdom | 16 (8) | 13 (8) | 10 (11) |
| **Ethnicity, n (%)** | |  |  |  |
|  | Asian | 98 (52) | 92 (54) | 44 (48) |
|  | White | 53 (28) | 47 (28) | 29 (32) |
|  | Mixed | 14 (7) | 14 (8) | 9 (10) |
|  | Black | 12 (6) | 7 (4) | 3 (4) |
|  | Hispanic | 5 (3) | 5 (3) | 3 (4) |
|  | Aboriginal or Torres Strait Islander | 2 (1) | 0 (0) | 2 (2) |
|  | Other | 4 (2) | 3 (2) | 1 (1) |
|  | Prefer not to say | 2 (1) | 1 (1) | 0 (0) |
| **Level of Education,** **n (%)** | |  |  |  |
|  | High School | 49 (26) | 41 (24) | 24 (26) |
|  | Professional / vocational training | 7 (4) | 5 (3) | 6 (7) |
|  | University | 133 (70) | 122 (72) | 60 (66) |
|  | Prefer not to say | 1 (1) | 1 (1) | 1 (1) |
| **Perceived Wealth, n (%)** | |  |  |  |
|  | Not at all | 9 (5) | 8 (5) | 6 (7) |
|  | Not very | 34 (18) | 31 (18) | 17 (19) |
|  | Fairly | 90 (47) | 80 (47) | 46 (50) |
|  | Rather | 40 (21) | 36 (21) | 16 (18) |
|  | Very | 10 (5) | 8 (5) | 3 (3) |
|  | Prefer not to say | 7 (4) | 6 (4) | 3 (3) |
| **Source, n (%)** | |  | |  |
|  | UNSW Students – 1^st^ year^a^ | 100 (53) | 92 (54) | 35 (39 ) |
|  | Social Media^b^ | 47 (25) | 38 (22) | 22 (24) |
|  | Prolific | 20 (11) | 19 (11) | 15 (16) |
|  | UNSW - Other Students & Alumni | 19 (10) | 18 (11) | 16 (18) |
|  | Other^c^ | 4 (3) | 2 (2) | 3 (3) |

^a^UNSW Students – 1^st^ year are samples recruited from UNSW first year psychology students in return for course credit. ^b^Social Media included participants recruited from advertising on Facebook and Instagram. ^c^Other participants were sourced via word-of-mouth.

**Figure S1.** Participant recruitment and engagement.

**Table S2.** Attrition analysis: comparison of completers and non-completers on baseline variables.

| Variable | Non-Completers^a^  M (SD) | Completers  M (SD) ^a^ | *t* | *df* | *P* |
| --- | --- | --- | --- | --- | --- |
| Anxiety^b^ | 8.30 (4.80) | 7.63 (4.78) | 0.86 | 188 | .390 |
| Depression^b^ | 8.08 (5.14) | 7.85 (4.99) | 0.29 | 188 | .773 |
| Social Sensitivity^b^ | -0.08 (0.99) | 0.09 (0.98) | -1.22 | 187 | .224 |
| Smartphone Time^c^ | -0.07 (0.99) | 0.07 (1.01) | -0.88 | 166 | .379 |
| Social Media Time^c^ | 0.00 (1.00) | -0.01 (1.01) | 0.06 | 147 | .951 |
| Texting Time^c^ | 0.01 (1.00) | -0.01 (1.00) | 0.16 | 152 | .874 |
| EMA Compliance^d^ | 0.54 (0.31) | 0.76 (0.27) | -4.69 | 151 | **< .001** |

^a^Non-completers = participants who did not complete the 1-month follow-up (n = 99); Completers = participants who completed the 1-month follow-up (n = 91).

^b^Anxiety=total score on the 7-item Generalized Anxiety Disorder Scale (GAD-7)^1^; Depression=total score on the 8-item Patient Health Questionnaire (PHQ-8)^2^; Social Sensitivity= total score on the Online and Offline Social Sensitivity Questionnaire (O^2^S^3^)^3^.

^c^Smartphone time, social media time and texting time is operationalized as average daily minutes spent on smartphones and social media and texting apps, respectively, extracted from participant screenshots. Online social interaction time is combined social media and texting time. All time variables are standardised (z-scored).

^d^EMA Compliance = proportion of 21 EMA surveys completed.

**Table S3.** Online social interaction applications list.

| Communication Type | Smartphone Applications |
| --- | --- |
| Social Media | - Facebook - Instagram - TikTok - X (formerly known as Twitter) - SnapChat - LinkedIn - Discord |
| Texting | - FB Messenger - WhatsApp - Messages - QQ - Telegram - WeChat |

**Table S4.** Sensitivity analysis: cross-sectional associations between responses and mental health symptoms for full and compliance-restricted samples (≥50% ema compliance).

|  |  | Full Sample  (N=166) | | | Compliance Restricted^a^  (N=111) | | |
| --- | --- | --- | --- | --- | --- | --- | --- |
| Predictor | | *F* test (df) | *P^b^* | η²*_p_^c^* | *F* test (df) | *P^b^* | η²*_p_^c^* |
| **Affective Response^d^** | |  |  |  |  |  |  |
|  | EMA^e^ | 1.37 (20,1453) | 0.13 | .02 | 1.21 (20,1278) | 0.24 | .02 |
|  | Anxiety | 6.73 (1,155) | 0.01 | .04 | 6.94 (1,104) | 0.01 | .06 |
|  | Depression | 3.18 (1,157) | 0.08 | .02 | 4.17 (1,104) | 0.04 | .04 |
| **Affiliative Response^d^** | |  |  |  |  |  |  |
|  | EMA^e^ | 1.66 (20,1439) | 0.03 | .02 | 1.46 (20,1275) | 0.08 | .02 |
|  | Anxiety | 3.37 (1,159) | 0.07 | .02 | 3.41 (1,108) | 0.07 | .03 |
|  | Depression | 1.35 (1,161) | 0.25 | .01 | 2.42 (1,108) | 0.12 | .02 |

^a^Compliance restricted = sensitivity sample restricted to participants completing ≥50% of EMA surveys (all connection types; n = 112, 66.3% of EMA sample).

^b^ Alpha threshold set to .025 applying a Bonferroni correction for the two outcomes of interest (anxiety and depression symptoms).

^c^η ^2^_p_ is partial eta-squared, where .01, .06 and .14 are small, medium, and large effect sizes, respectively^4^.

^d^At each EMA participants reported their affective and affiliative response to their most important social interaction in the past two hours on a scale ranging from 0=*Very Unhappy* to 100=*Very Happy* and from 0=*Left out* to 100=*Included*, respectively.

^g^21 EMA timepoints: 3/day for 7 days.

^f^Anxiety=total score on the 7-item Generalized Anxiety Disorder Scale (GAD-7)^1^; Depression=total score on the 8-item Patient Health Questionnaire (PHQ-8)^2^.

**Table S5.** Sensitivity analysis: EMA-level linear mixed model associations between connection type and responses moderated by mental health symptoms for full and compliance-restricted samples (≥50% ema compliance).

|  | | Full Sample  (*N*=166) | | | Compliance Restricted^a^  (*N*=111) | | |
| --- | --- | --- | --- | --- | --- | --- | --- |
| Predictor | | *F* test (df) | *P^b^* | η²*_p_^c^* | *F* test (df) | *P^b^* | η²*_p_^c^* |
| **Affective Response^d^** | | | | | | | |
| Connection Type^e^ | | 10.12 (3,1532) | < .001 | .02 | 10.54 (3,1345) | < .001 | .02 |
| **Anxiety^f^** | |  |  |  |  |  |  |
|  | EMA^g^ | 1.27 (20,1449) | 0.19 | .02 | 1.15 (20,1274) | 0.29 | .02 |
|  | Anxiety | 4.53 (1,269) | 0.03 | .02 | 5.15 (1,162) | 0.02 | .03 |
|  | Connection Type | 10.12 (3,1532) | < .001 | .02 | 10.54 (3,1345) | < .001 | .02 |
|  | Anxiety x Connection Type | 0.71 (3,1536) | 0.55 | .00 | 0.78 (3,1351) | 0.50 | .00 |
| **Depression^f^** | |  |  |  |  |  |  |
|  | EMA^g^ | 1.31 (20,1448) | 0.16 | .02 | 1.19 (20,1273) | 0.26 | .02 |
|  | Depression | 0.53 (1,258) | 0.47 | .00 | 1.22 (1,159) | 0.27 | .01 |
|  | Connection Type | 9.79 (3,1531) | < .001 | .02 | 10.20 (3,1344) | < .001 | .02 |
|  | Depression x Connection Type | 1.61 (3,1538) | 0.19 | .00 | 1.38 (3,1352) | 0.25 | .00 |
| **Affiliative Response^d^** | | | | | | | |
| Connection Type^e^ | | 8.16 (3,1502) | < .001 | .02 | 8.35 (3,1322) | < .001 | .02 |
| **Anxiety^f^** | |  |  |  |  |  |  |
|  | EMA^g^ | 1.60 (20,1435) | 0.05 | .02 | 1.41 (20,1270) | 0.11 | .02 |
|  | Anxiety | 3.21 (1,237) | 0.07 | .01 | 3.34 (1,145) | 0.07 | .02 |
|  | Connection Type | 8.16 (3,1502) | < .001 | .02 | 8.35 (3,1322) | < .001 | .02 |
|  | Anxiety x Connection Type | 0.29 (3,1504) | 0.83 | .00 | 0.42 (3,1327) | 0.74 | .00 |
| **Depression^f^** | |  |  |  |  |  |  |
|  | EMA^g^ | 1.63 (20,1433) | 0.04 | .02 | 1.44 (20,1270) | 0.09 | .02 |
|  | Depression | 0.50 (1,231) | 0.48 | .00 | 1.23 (1,145) | 0.27 | .01 |
|  | Connection Type | 7.67 (3,1502) | < .001 | .02 | 7.92 (3,1322) | < .001 | .02 |
|  | Depression x Connection Type | 0.73 (3,1514) | 0.53 | .00 | 0.51 (3,1331) | 0.68 | .00 |

^a^Compliance restricted = sensitivity sample restricted to participants completing ≥50% of EMA surveys (all connection types; n = 112, 66.3% of EMA sample).

^b^ Alpha threshold set to .025 applying a Bonferroni correction for the two outcomes of interest (anxiety and depression symptoms).

^c^η ^2^_p_ is partial eta-squared, where .01, .06 and .14 are small, medium, and large effect sizes, respectively^4^.

^d^At each EMA participants reported their affective and affiliative response to their most important social interaction in the past two hours on a scale ranging from 0=*Very Unhappy* to 100=*Very Happy* and from 0=*Left out* to 100=*Included*, respectively.

^e^Connection types = In-person and online social interaction (social media and texting) apps.

^f^Anxiety=total score on the 7-item Generalized Anxiety Disorder Scale (GAD-7)^1^; Depression=total score on the 8-item Patient Health Questionnaire (PHQ-8)^2^.

^g^21 EMA timepoints: 3/day for 7 days.

**Table S6.** Sensitivity analysis: associations between responses and mental health symptoms- longitudinal model results for full and compliance-restricted samples (≥50% compliance).

|  | | Full Sample  (*N*=169) | | | Compliance Restricted^a^  (*N*=112) | | |
| --- | --- | --- | --- | --- | --- | --- | --- |
| Predictor | | *F* test (*df*) | *P* | η²_p_^b^ | *F* test (*df*) | *P* | η²_p_^b^ |
| **Anxiety^c^** | | | | | | | |
| **Affective Response^d^** | |  |  |  |  |  |  |
|  | Affective Response | 6.86 (1,176) | .01 | .04 | 8.04 (1,115) | .01 | .07 |
|  | Timepoint**^e^** | 2.47 (1,96) | .12 | .03 | 0.73 (1,74) | .40 | .01 |
|  | Timepoint x Affective Response | 0.41 (1,96) | .53 | .00 | 0.65 (1,75) | .42 | .01 |
| **Affiliative Response^d^** | |  |  |  |  |  |  |
|  | Affiliative Response | 3.38 (1,169) | .07 | .02 | 3.46 (1,109) | .07 | .03 |
|  | Timepoint**^d^** | 2.22 (1,96) | .14 | .02 | 0.75 (1,74) | .39 | .01 |
|  | Timepoint x Affiliative Response | 0.07 (1,94) | .79 | .00 | 0.02 (1,73) | .89 | .00 |
| **Depression^c^** | | | | | | | |
| **Affective Response^d^** | |  |  |  |  |  |  |
|  | Affective Response | 4.13 (1,175) | .04 | .02 | 4.18 (1,115) | .04 | .03 |
|  | Timepoint**^e^** | 0.04 (1,96) | .84 | .00 | 0.01 (1,76) | .94 | .00 |
|  | Timepoint x Affective Response | 1.73 (1,96) | .19 | .02 | 0.25 (1,77) | .62 | .00 |
| **Affiliative Response^d^** | |  |  |  |  |  |  |
|  | Affiliative Response | 3.49 (1,166) | .06 | .02 | 3.31 (1,109) | .07 | .03 |
|  | Timepoint**^d^** | 0.18 (1,95) | .68 | .00 | 0.02 (1,76) | .88 | .00 |
|  | Timepoint x Affiliative Response | 3.28 (1,93) | .07 | .03 | 0.38 (1,74) | .54 | .01 |

^a^Compliance restricted = sensitivity sample restricted to participants completing ≥50% of EMA surveys (n = 112, 66.3% of EMA sample).

^b^η ^2^_p_ is partial eta-squared, where .01, .06 and .14 are small, medium, and large effect sizes, respectively^4^.

^c^ Anxiety=total score on the 7-item Generalized Anxiety Disorder Scale (GAD-7)^1^; Depression=total score on the 8-item Patient Health Questionnaire (PHQ-8)^2^.

^d^At each EMA participants reported their affective and affiliative response to their most important social interaction in the past two hours on a scale ranging from 0=Very Unhappy to 100=Very Happy and from 0=Left out to 100=Included, respectively.

^e^Timepoint*=*baseline vs. 1-month follow-up.

Table S7. Relationship between average daily social media and texting time and mental health at baseline.

|  | Anxiety^a^ | | | | |  | Depression^a^ | | | | |
| --- | --- | --- | --- | --- | --- | --- | --- | --- | --- | --- | --- |
| Predictor | *β* | *SE* | *t* | *P* | η^2^*_p_* |  | *β* | *SE* | *t* | *P* | η^2^*_p_* |
| Social Media Time^b^ | 0.23 | 0.08 | 2.94 | .00 | .05 |  | 0.14 | 0.08 | 1.76 | .08 | .02 |
| Texting Time^b^ | 0.21 | 0.08 | 2.69 | .01 | .05 |  | 0.15 | 0.08 | 1.82 | .07 | .02 |

^a^Anxiety=total score on the 7-item Generalized Anxiety Disorder Scale (GAD-7)^1^; Depression=total score on the 8-item Patient Health Questionnaire (PHQ-8)^2^; Social Sensitivity= total score on the Online and Offline Social Sensitivity Questionnaire (O^2^S^3^)^3^.

^b^Social media time and texting time is operationalized as average daily minutes spent on social media and texting apps, respectively, extracted from participant screenshots standardised (z-scored).

Table S8. Relationship between average daily screentime, by type, and mental health, over time (baseline and 1-month follow-up).

|  |  | Anxiety^a^ | | | | Depression^a^ | | | |
| --- | --- | --- | --- | --- | --- | --- | --- | --- | --- |
| Predictor | | *F* test *(df)* | | *P* | η^2^*_p_^b^* | *F* test (*df)* | | *P* | η^2^*_p_^b^* |
| **Smartphone Time^c^** | |  |  |  |  |  |  |  |  |
|  | Timepoint^d^ | 3.02 (1, 98) | | .09 | .03 | 0.00 (1, 101) | | .95 | .00 |
|  | Smartphone time | 0.11 (1, 174) | | .74 | .00 | 0.03 (1, 175) | | .87 | .00 |
|  | Smartphone time x Timepoint | 0.12 (1, 96) | | .73 | .00 | 0.25 (1, 99) | | .62 | .00 |
| **Social Media Time^c^** | |  |  |  |  |  |  |  |  |
|  | Timepoint^d^ | 2.58 (1, 88) | | .11 | .03 | 0.02 (1, 91) | | .88 | .00 |
|  | Social media time | 5.25 (1, 202) | | .02 | .03 | 3.23 (1, 204) | | .07 | .02 |
|  | Social media time x Timepoint | 0.75 (1, 95) | | .39 | .01 | 0.01 (1, 98) | | .91 | .00 |
| **Texting Time^c^** | |  |  |  |  |  |  |  |  |
|  | Timepoint^d^ | 2.93 (1, 86) | | .09 | .03 | 0.03 (1, 84) | | .86 | .00 |
|  | Texting time | 4.86 (1, 175) | | .03 | .03 | 0.31 (1, 175) | | .58 | .00 |
|  | Texting time x Timepoint | 0.77 (1, 88) | | .38 | .01 | 4.76 (1, 86) | | .03 | .05 |
| **Total Online Social Interaction (OSI) Time^c^** | |  |  |  |  |  |  |  |  |
|  | Timepoint^d^ | 2.35 (1, 82) | | .13 | .03 | 0.07 (1, 79) | | .80 | .00 |
|  | OSI time | 7.07 (1, 188) | | .01 | .04 | 3.09 (1, 187) | | .08 | .02 |
|  | OSI time x Timepoint | 1.72 (1, 87) | | .19 | .02 | 0.49 (1, 84) | | .49 | .01 |

^a^Anxiety=total score on the 7-item Generalized Anxiety Disorder Scale (GAD-7)^1^; Depression=total score on the 8-item Patient Health Questionnaire (PHQ-8)^2^.

^b^η ^2^_p_ is partial eta-squared, where .01, .06 and .14 are small, medium, and large effect sizes, respectively^4^.

^c^Smartphone time, social media time and texting time is operationalized as average daily minutes spent on smartphones and social media and texting apps, respectively, extracted from participant screenshots. Online social interaction time is combined social media and texting time. All time variables are standardised (z-scored).

^d^Timepoint*=*baseline vs. 1-month follow-up.

Table S9. Descriptives of social interactions in 2 hours prior to ema prompt.

| Variable | | Value | |
| --- | --- | --- | --- |
| **Responses with a Social Interaction, n (%)** | |  |  |
|  | Yes | 1,609 (70) | |
|  | No | 673 (29) | |
|  | Not completed | 22 | 1 |
| **Average Number of Total Interactions** | |  |  |
|  | In-person | 3.2 | 61 |
|  | Online | 2.8 | 39 |
|  | Total | 4.5 | 100 |
| **Most Important Social Interaction** | | | |
| **Connection Type** | |  |  |
|  | In-person | 1,078 | 67 |
|  | Phone / Video | 193 | 12 |
|  | Social Media | 75 | 5 |
|  | Texting | 249 | 16 |
|  | Gaming / Other | 6 | 0.4 |
| **Type of Interaction** | |  |  |
|  | One-way | 110 | 7 |
|  | Two-way | 1,166 | 73 |
|  | Private Group | 268 | 17 |
|  | Public | 53 | 3 |
| **People Involved in the Interaction** | |  |  |
|  | Spouse / Partner | 308 | 19 |
|  | Friend | 637 | 40 |
|  | Family | 512 | 32 |
|  | Acquaintance / Colleague / Classmate Neighbor | 241 | 15 |
|  | Boss / Teacher | 35 | 2 |
|  | Someone you don’t know | 112 | 7 |
| **Initiator of the Interaction** | |  |  |
|  | Me | 754 | 47 |
|  | Someone else | 841 | 53 |

**Table S10.** Relationship between connection type and momentary affective and affiliative responses for social media and texting.

|  | Affective Response^a^ | | | | | Affiliative Response^a^ | | | | |
| --- | --- | --- | --- | --- | --- | --- | --- | --- | --- | --- |
| Predictor | *β* | *SE* | *t* | *P* | CI | *β* | *SE* | *t* | *P* | CI |
| Social Media | -6.45 | 2.25 | -2.87 | .004 | -10.85 to -2.05 | -7.45 | 2.12 | -3.52 | <.001 | -11.60 to -3.30 |
| Texting | -6.62 | 1.34 | -4.93 | <.001 | -9.25 to -3.99 | -4.85 | 1.23 | -3.83 | <.001 | -7.33 to -2.37 |

^a^At each EMA participants reported their affective and affiliative response to their most important social interaction in the past two hours on a scale ranging from 0=*Very Unhappy* to 100=*Very Happy* and from 0=*Left out* to 100=*Included*, respectively.

**Table S11.** Relationship between connection type and momentary affective and affiliative responses, moderated by baseline anxiety and depression.

|  | | Affective Response^a^ | | | | | Affiliative Response^a^ | | | | |
| --- | --- | --- | --- | --- | --- | --- | --- | --- | --- | --- | --- |
| Predictor | | β | *SE* | *t* | *P* | 95% CI | β | *SE* | *t* | *P* | 95% CI |
| **Anxiety^b^** | |  |  |  |  |  |  |  |  |  |  |
|  | Anxiety | -2.65 | 1.02 | -2.60 | 0.01 | -4.66 to  -0.65 | -2.24 | 1.19 | -1.88 | .06 | -4.57 to 0.10 |
|  | Connection Type^c^ | -6.47 | 1.21 | -5.34 | <.001 | -8.84 to  -4.09 | -5.50 | 1.14 | -4.81 | <.001 | -7.74 to  -3.26 |
|  | Anxiety x Connection Type | 1.24 | 1.31 | 0.95 | .34 | -1.32 to 3.80 | 0.52 | 1.24 | 0.42 | .67 | -1.90 to 2.95 |
| **Depression^b^** | |  |  |  |  |  |  |  |  |  |  |
|  | Depression | -2.56 | 1.04 | -2.46 | .01 | -4.60 to -0.52 | -0.34 | 0.22 | -1.58 | 0.12 | -0.76 to 0.08 |
|  | Connection Type^c^ | -6.44 | 1.21 | -5.32 | <.001 | -8.81 to -4.07 | -6.08 | 2.02 | -3.01 | 0.003 | -10.03 to -2.12 |
|  | Depression x Connection Type | 1.49 | 2.29 | 1.16 | .25 | -1.03 to 4.01 | 0.07 | 0.22 | 0.34 | .74 | -0.36 to 0.50 |

^a^At each EMA participants reported their affective and affiliative response to their most important social interaction in the past two hours on a scale ranging from 0=*Very Unhappy* to 100=*Very Happy* and from 0=*Left out* to 100=*Included*, respectively.

^b^Anxiety=total score on the 7-item Generalized Anxiety Disorder Scale (GAD-7)^1^; Depression=total score on the 8-item Patient Health Questionnaire (PHQ-8)^2^.

^c^ In-person and via online social interaction (social media and texting) apps.

**Table S12.** Relationship between affective and affiliative responses to social interactions and mental health over time (baseline and one-month), for all interactions and by connection type.

|  |  | Anxiety^a^ | | | | Depression^a^ | | | |  |
| --- | --- | --- | --- | --- | --- | --- | --- | --- | --- | --- |
|  |  | *F* test (*df*) | | *P^b^* | η^2^*_p_^c^* | *F* test (*df*) | | *P^b^* | η^2^*_p_^c^* |  |
| a. **All Interactions^d^** | | | | | | | | | |  |
| **Affective Response^e^** | |  |  |  |  |  |  |  |  |  |
|  | Timepoint^f^ | 2.47 (1, 96) | | .12 | .03 | 0.04 (1, 96) | | .839 | .00 |  |
|  | Affective Response | 6.86 (1, 176) | | .01 | .04 | 4.13 (1, 175) | | .044 | .02 |  |
|  | Affective Response x Timepoint | 0.41 (1, 96) | | .53 | .00 | 1.73 (1, 96) | | .191 | .02 |  |
| **Affiliative Response^e^** | |  |  |  |  |  |  |  |  |  |
|  | Timepoint^f^ | 2.22 (1, 96) | | .14 | .02 | 0.18 (1, 95) | | .676 | .00 |  |
|  | Affiliative Response | 3.38 (1, 183) | | .07 | .02 | 3.49 (1, 166) | | .064 | .02 |  |
|  | Affiliative Response x Timepoint | 0.07 (1, 94) | | .79 | .00 | 3.28 (1, 93) | | .073 | .03 |  |
| b. **In-Person Interactions** | | | | | | | | | |  |
| **Affective Response^e^** | |  |  |  |  |  |  |  |  |  |
|  | Timepoint^f^ | 2.29 (1,92) | | .13 | .02 | 0.08 (1,93) | | .77 | .00 |  |
|  | Affective Response | 4.63 (1,158) | | .03 | .03 | 7.39 (1,157) | | .01 | .04 |  |
|  | Affective Response x Timepoint | 0.03 (1,92) | | .86 | .00 | 1.68 (1,93) | | .20 | .02 |  |
| **Affiliative Response^e^** | |  |  |  |  |  |  |  |  |  |
|  | Timepoint^f^ | 2.26 (1,92) | | .14 | .02 | 0.22 (1,93) | | .64 | .00 |  |
|  | Affiliative Response | 2.76 (1,158) | | .10 | .02 | 7.42 (1,158) | | .01 | .04 |  |
|  | Affiliative Response x Timepoint | 0.01 (1,92) | | .92 | .00 | 2.85 (1,93) | | .10 | .03 |  |
| c. **Phone /Video Interactions** | | | | | | | | | |  |
| **Affective Response^e^** | |  | |  |  |  | |  |  |  |
|  | Timepoint^f^ | 0.19 (1,55) | | .67 | .00 | 0.05 (1,59) | | .83 | .00 |  |
|  | Affective Response | 4.25 (1,83) | | .04 | .05 | 0.01 (1,84) | | .92 | .00 |  |
|  | Affective Response x Timepoint | 0.64 (1,52) | | .43 | .01 | 0.45 (1,55) | | .51 | .01 |  |
| **Affiliative Response^e^** | |  | |  |  |  | |  |  |  |
|  | Timepoint^f^ | 0.07 (1,56) | | .79 | .00 | 0.07 (1,59) | | .79 | .00 |  |
|  | Affiliative Response | 6.14 (1,86) | | .02 | **.**07 | 1.28 (1,86) | | .26 | .01 |  |
|  | Affiliative Response x Timepoint | 0.11 (1,53) | | .74 | .00 | 0.00 (1,56) | | .99 | .00 |  |
| d. **Social Media Interactions** | | | | | | | | | |  |
| **Affective Response^e^** | |  |  |  |  |  |  |  |  |  |
|  | Timepoint^f^ | 0.22 (1,26) | | .64 | .01 | 0.17 (1,29) | | .68 | .01 |  |
|  | Affective Response | 1.73 (1,48) | | .12 | .03 | 0.05 (1,51) | | .82 | .00 |  |
|  | Affective Response x Timepoint | 2.74 (1,26) | | .11 | .10 | 2.26 (1,30) | | .14 | .07 |  |
| **Affiliative Response^e^** | |  |  |  |  |  |  |  |  |  |
|  | Timepoint^f^ | 0.27 (1,26) | | .61 | .01 | 0.14 (1,30) | | .71 | .00 |  |
|  | Affiliative Response | 1.93 (1,46) | | .17 | .04 | 0.00 (1,48) | | .97 | .00 |  |
|  | Affiliative Response x Timepoint | 0.65 (1,26) | | .43 | .02 | 0.82 (1,30) | | .37 | .03 |  |
| e. **Texting Interactions** | | | | | | | | | |  |
| **Affective Response^e^** | |  |  |  |  |  |  |  |  |  |
|  | Timepoint^f^ | 0.58 (1.65) | | .45 | .01 | 0.03 (1,67) | | .86 | .00 |  |
|  | Affective Response | 0.19 (1,104) | | .66 | .00 | 0.09 (1,105) | | .76 | .00 |  |
|  | Affective Response x Timepoint | 0.67 (1,66) | | .42 | .01 | 0.12 (1,67) | | .73 | .00 |  |
| **Affiliative Response^e^** | |  |  |  |  |  |  |  |  |  |
|  | Timepoint^f^ | 0.46 (1, 66) | | .50 | .01 | 0.13 (1,67) | | .71 | .00 |  |
|  | Affiliative Response | 0.55 (1,100) | | .46 | .01 | 4.33 (1,100) | | .04 | .04 |  |
|  | Affiliative Response x Timepoint | 0.37 (1,64) | | .54 | .01 | 0.98 (1,66) | | .33 | .01 |  |
| f. **Total Online Social Interactions** | | | | | | | | | |  |
| **Affective Response^e^** | |  |  |  |  |  |  |  |  |  |
|  | Timepoint^f^ | 0.47 (1,75) | | .50 | .01 | 0.04 (1,79) | | .85 | .00 |  |
|  | Affective Response | 0.53 (1,123) | | .47 | .00 | 0.03 (1,123 | | .87 | .00 |  |
|  | Affective Response x Timepoint | 0.87 (1,75) | | .35 | .01 | 0.16 (1,79) | | .69 | .00 |  |
| **Affiliative Response^e^** | |  |  |  |  |  |  |  |  |  |
|  | Timepoint^f^ | 0.36 (1,76) | | .55 | .00 | 0.16 (1,79) | | .69 | .00 |  |
|  | Affiliative Response | 1.23 (1,122) | | .27 | .01 | 3.20 (1,122) | | .08 | .03 |  |
|  | Affiliative Response x Timepoint | 0.25 (1,75) | | .62 | .00 | 1.83 (1,78) | | .18 | .02 |  |

^a^Anxiety=total score on the 7-item Generalized Anxiety Disorder Scale (GAD-7)^1^; Depression=total score on the 8-item Patient Health Questionnaire (PHQ-8)^2^.

^b^ Alpha threshold set to .025 applying a Bonferroni correction for the two outcomes of interest (anxiety and depression symptoms).

^c^η ^2^_p_ is partial eta-squared, where .01, .06 and .14 are small, medium, and large effect sizes, respectively^4^.

^d^All interactions includes all in-person, phone, social media and texting interactions.

^e^At each EMA participants reported their affective and affiliative response to their most important social interaction in the past two hours on a scale ranging from 0=*Very Unhappy* to 100=*Very Happy* and from 0=*Left out* to 100=*Included*, respectively.

^f^Timepoint*=*baseline vs. 1-month follow-up.

**Table S13.** Baseline association between affective and affiliative response, and anxiety and depression, moderated by social sensitivity for all interactions and by connection type.

|  |  | Anxiety^a^ | | | |  | Depression^a^ | | | |
| --- | --- | --- | --- | --- | --- | --- | --- | --- | --- | --- |
| Predictor | | *F* test | *df* | *P^b^* | η^2^_p_*^c^* |  | *F* test | *df* | *P* | η^2^ _p_*^c^* |
|  | a. **All Interactions^d^** | | | | | | | | | |
| **Affective Response^e^** | |  |  |  |  |  |  |  |  |  |
|  | Affective Response | 5.66 | 1,160 | .02 | .03 |  | 1.73 | 1,160 | .19 | .01 |
|  | Social Sensitivity^f^ | 37.90 | 1,160 | <.001 | .19 |  | 33.33 | 1,160 | <.001 | .17 |
|  | Affective Response x Social Sensitivity | 1.35 | 1,160 | .25 | .01 |  | 0.61 | 1,160 | .44 | .00 |
| **Affiliative Response** | |  |  |  |  |  |  |  |  |  |
|  | Affiliative Response | 3.11 | 1,160 | .08 | .02 |  | 0.76 | 1,160 | .39 | .00 |
|  | Social Sensitivity | 40.13 | 1,160 | <.001 | .20 |  | 34.87 | 1,160 | <.001 | .18 |
|  | Affiliative Response x Social Sensitivity | 0.02 | 1,160 | .89 | .00 |  | 2.60 | 1,160 | .11 | .02 |
|  | b. **In-Person Interactions** | | | | | | | | | |
| **Affective Response** | |  |  |  |  |  |  |  |  |  |
|  | Affective Response | 4.47 | 1, 147 | .04 | .03 |  | 3.99 | 1, 147 | .05 | .03 |
|  | Social Sensitivity | 32.52 | 1, 147 | < .001 | .18 |  | 27.09 | 1, 147 | < .001 | .16 |
|  | Affective Response x Social Sensitivity | 0.15 | 1, 147 | .70 | .00 |  | 1.24 | 1, 147 | .27 | .01 |
| **Affiliative Response** | |  |  |  |  |  |  |  |  |  |
|  | Affiliative Response | 3.18 | 1, 147 | .08 | .02 |  | 3.23 | 1, 147 | .07 | .02 |
|  | Social Sensitivity | 33.31 | 1, 147 | < .001 | .18 |  | 27.85 | 1, 147 | < .001 | .16 |
|  | Affiliative Response x Social Sensitivity | 0.75 | 1, 147 | .39 | .01 |  | 2.67 | 1, 147 | .11 | .02 |
|  | c. **Phone Interactions** | | | | | | | | | |
| **Affective Response** | |  |  |  |  |  |  |  |  |  |
|  | Affective Response | 3.12 | 1, 80 | .08 | .04 |  | 0.28 | 1, 80 | .60 | .00 |
|  | Social Sensitivity | 38.42 | 1, 80 | < .001 | .32 |  | 23.04 | 1, 80 | < .001 | .22 |
|  | Affective Response x Social Sensitivity | 1.30 | 1, 80 | .26 | .02 |  | 0.11 | 1, 80 | .74 | .00 |
| **Affiliative Response** | |  |  |  |  |  |  |  |  |  |
|  | Affiliative Response | 6.33 | 1, 80 | .01 | .07 |  | 1.39 | 1, 80 | .24 | .02 |
|  | Social Sensitivity | 37.17 | 1, 80 | < .001 | .32 |  | 22.89 | 1, 80 | < .001 | .22 |
|  | Affiliative Response x Social Sensitivity | 1.32 | 1, 80 | .25 | .02 |  | 0.00 | 1, 80 | .99 | .00 |
| d. **Social Media Interactions** | | | | | | | | | | |
| **Affective Response** | |  |  |  |  |  |  |  |  |  |
|  | Affective Response | 0.40 | 1,39 | .53 | .01 |  | 0.56 | 1,39 | .46 | .01 |
|  | Social Sensitivity | 9.74 | 1,39 | .00 | .20 |  | 10.41 | 1,39 | .00 | .21 |
|  | Affective Response x Social Sensitivity | 0.40 | 1,39 | .53 | .01 |  | 6.84 | 1,39 | .01 | .15 |
| **Affiliative Response** | |  |  |  |  |  |  |  |  |  |
|  | Affiliative Response | 1.18 | 1,39 | .28 | .03 |  | 0.28 | 1,39 | .60 | .01 |
|  | Social Sensitivity | 9.85 | 1,39 | .00 | .20 |  | 10.14 | 1,39 | .00 | .21 |
|  | Affiliative Response x Social Sensitivity | 1.61 | 1,39 | .21 | .04 |  | 6.77 | 1,39 | .01 | .15 |
|  | e. **Texting Interactions** | | | | | | | | | |
|  | **Affective Response** |  |  |  |  |  |  |  |  |  |
|  | Affective Response | 0.00 | 1, 94 | .96 | .00 |  | 0.29 | 1, 94 | .59 | .00 |
|  | Social Sensitivity | 25.05 | 1, 94 | < .001 | .21 |  | 23.27 | 1, 94 | < .001 | .20 |
|  | Affective Response x Social Sensitivity | 0.52 | 1, 94 | .47 | .01 |  | 0.02 | 1, 94 | .89 | .00 |
|  | **Affiliative Response** |  |  |  |  |  |  |  |  |  |
|  | Affiliative Response | 0.16 | 1, 94 | .69 | .00 |  | 2.24 | 1, 94 | .14 | .02 |
|  | Social Sensitivity | 24.14 | 1, 94 | < .001 | .20 |  | 22.88 | 1, 94 | < .001 | .20 |
|  | Affiliative Response x Social Sensitivity | 0.25 | 1, 94 | .62 | .00 |  | 0.09 | 1, 94 | .77 | .00 |
|  | f. **Total Online Social Interactions** | | | | | | | | | |
|  | **Affective Response** |  |  |  |  |  |  |  |  |  |
|  | Affective Response | 0.03 | 1, 111 | .86 | .00 |  | 0.01 | 1, 111 | .92 | .00 |
|  | Social Sensitivity | 25.01 | 1, 111 | < .001 | .18 |  | 24.78 | 1, 111 | < .001 | .18 |
|  | Affective Response x Social Sensitivity | 0.30 | 1, 111 | .59 | .00 |  | 0.24 | 1, 111 | .63 | .00 |
|  | **Affiliative Response** |  |  |  |  |  |  |  |  |  |
|  | Affiliative Response | 0.69 | 1, 111 | .41 | .01 |  | 0.87 | 1, 111 | .35 | .01 |
|  | Social Sensitivity | 23.93 | 1, 111 | < .001 | .18 |  | 23.16 | 1, 111 | < .001 | .17 |
|  | Affiliative Response x Social Sensitivity | 0.01 | 1, 111 | .92 | .00 |  | 0.12 | 1, 111 | .73 | .00 |

^a^Anxiety=total score on the 7-item Generalized Anxiety Disorder Scale (GAD-7)^1^; Depression=total score on the 8-item Patient Health Questionnaire (PHQ-8)^2^.

^b^ Alpha threshold set to .025 applying a Bonferroni correction for the two outcomes of interest (anxiety and depression symptoms).

^c^η ^2^_p_ is partial eta-squared, where .01, .06 and .14 are small, medium, and large effect sizes, respectively^4^.

^d^All interactions includes all in-person, phone, social media and texting interactions.

^e^At each EMA participants reported their affective and affiliative response to their most important social interaction in the past two hours on a scale ranging from 0=*Very Unhappy* to 100=*Very Happy* and from 0=*Left out* to 100=*Included*, respectively.

^f^Social Sensitivity= total score on the Online and Offline Social Sensitivity Questionnaire (O^2^S^3^)^3^.

**Table S14.** One-month association between affective and affiliative response, and anxiety and depression, moderated by social sensitivity for all interactions and by connection type.

|  |  | Anxiety^a^ | | | | Depression^a^ | | | |
| --- | --- | --- | --- | --- | --- | --- | --- | --- | --- |
|  | Predictor | *F* | *df* | *P^b^* | η^2^_p_^c^ | *F* | *df* | *P^b^* | η^2^ _p_^c^ |
| **All Interactions^d^** | | | | | | | | | |
| **Affective Response**^e^ | |  |  |  |  |  |  |  |  |
|  | Timepoint^f^ | 2.81 | 1,99 | .10 | .03 | 0.00 | (1,99) | .97 | .00 |
|  | Affective Response | 3.78 | 1,176 | .05 | .02 | 1.44 | (1,174) | .23 | .01 |
|  | Social Sensitivity^g^ | 35.42 | 1,182 | <.001 | .16 | 29.34 | (1,181) | <.001 | .14 |
|  | Timepoint x Affective Response | 0.39 | 1,99 | .53 | .00 | 2.12 | (1,99) | .15 | .02 |
|  | Timepoint x Social Sensitivity | 0.74 | 1,101 | .39 | .01 | 0.69 | (1,101) | .41 | .01 |
|  | Affective Response x Social Sensitivity | 1.12 | 1,174 | .29 | .01 | 0.40 | (1,173) | .53 | .00 |
|  | Timepoint v Affective Response x Social Sensitivity | 0.08 | 1,99 | .77 | .00 | 0.09 | (1,99) | .77 | .00 |
| **Affiliative Response**^e^ | |  |  |  |  |  |  |  |  |
|  | Timepoint^f^ | 2.69 | 1,100 | .10 | .03 | 0.09 | (1,100) | .77 | .00 |
|  | Affiliative Response | 2.33 | 1,169 | .13 | .01 | 2.55 | (1,167) | .11 | .02 |
|  | Social Sensitivity^g^ | 37.07 | 1,184 | <.001 | .17 | 29.82 | (1,183) | <.001 | .14 |
|  | Timepoint x Affiliative Response | 0.02 | 1,97 | .88 | .00 | 3.41 | (1,97) | .07 | .03 |
|  | Timepoint x Social Sensitivity | 0.64 | 1,101 | .43 | .01 | 0.81 | (1,102) | .37 | .01 |
|  | Affiliative Response x Social Sensitivity | 0.04 | 1, 170 | .84 | .00 | 2.24 | (1,168) | .14 | .01 |
|  | Timepoint x Affective Response x Social Sensitivity | 0.01 | 1,98 | .92 | .00 | 0.13 | (1,98) | .72 | .00 |
| **In-Person Interactions** | | | | | | | | | |
| **Affective Response**^e^ | |  |  |  |  |  |  |  |  |
|  | Timepoint^f^ | 2.43 | 1, 91 | .12 | .03 | 0.03 | (1, 92) | .86 | .00 |
|  | Affective Response | 2.45 | 1, 152 | .12 | .02 | 4.82 | (1, 150) | .03 | .03 |
|  | Social Sensitivity^g^ | 30.67 | 1, 162 | <.001 | .16 | 23.01 | (1, 161) | <.001 | .13 |
|  | Timepoint x Affective Response | 0.05 | 1, 90 | .82 | .00 | 2.03 | (1, 91) | .16 | .02 |
|  | Time x Social Sensitivity | 0.44 | 1, 93 | .51 | .00 | 0.57 | (1, 94) | .45 | .01 |
|  | Affective Response x Social Sensitivity | 0.08 | 1, 151 | .77 | .00 | 1.02 | (1, 149) | .32 | .01 |
|  | Time v Affective Response x Social Sensitivity | 0.04 | 1, 90 | .85 | .00 | 0.08 | (1, 91) | .78 | .00 |
| **Affiliative Response**^e^ | |  |  |  |  |  |  |  |  |
|  | Timepoint^f^ | 2.54 | 1, 92 | .12 | .03 | 0.17 | (1, 93) | .68 | .00 |
|  | Affiliative Response | 1.55 | 1, 152 | .22 | .01 | 6.89 | (1, 151) | .01 | .04 |
|  | Social Sensitivity^g^ | 30.59 | 1, 162 | <.001 | .16 | 22.23 | (1, 161) | <.001 | .12 |
|  | Timepoint x Affiliative Response | 0.02 | 1, 91 | .88 | .00 | 2.75 | (1, 91) | .10 | .03 |
|  | Time x Social Sensitivity | 0.38 | 1, 94 | .54 | .00 | 0.88 | (1, 94) | .35 | .01 |
|  | Affiliative Response x Social Sensitivity | 0.68 | 1, 154 | .41 | .00 | 2.95 | (1, 153) | .09 | .02 |
|  | Timepoint x Affective Response x Social Sensitivity | 0.02 | 1, 91 | .88 | .00 | 0.00 | (1, 92) | .99 | .00 |
| **Phone Interactions** | | | | | | | | | |
| **Affective Response**^e^ | |  |  |  |  |  |  |  |  |
|  | Timepoint^f^ | 0.58 | 1, 58 | .45 | .01 | 0.00 | (1, 57) | .96 | .00 |
|  | Affective Response | 6.39 | 1, 77 | .01 | .08 | 0.02 | (1, 73) | .90 | .00 |
|  | Social Sensitivity^g^ | 39.76 | 1, 85 | < .001 | .32 | 26.17 | (1, 82) | < .001 | .24 |
|  | Timepoint x Affective Response | 0.19 | 1, 54 | .66 | .00 | 0.23 | (1, 52) | .63 | .00 |
|  | Timepoint x Social Sensitivity | 0.15 | 1, 58 | .70 | .00 | 0.00 | (1, 56) | .95 | .00 |
|  | Affective Response x Social Sensitivity | 0.21 | 1, 75 | .65 | .00 | 0.61 | (1, 71) | .44 | .01 |
|  | Timepoint v Affective Response x Social Sensitivity | 1.36 | 1, 53 | .25 | .03 | 0.30 | (1, 51) | .58 | .01 |
| **Affiliative Response**^e^ | |  |  |  |  |  |  |  |  |
|  | Timepoint^f^ | 0.27 | 1, 60 | .61 | .00 | 0.01 | (1, 59) | .93 | .00 |
|  | Affiliative Response | 6.17 | 1, 80 | .02 | .07 | 1.07 | (1, 77) | .31 | .01 |
|  | Social Sensitivity^g^ | 36.05 | 1, 86 | < .001 | .30 | 24.95 | (1, 83) | < .001 | .23 |
|  | Timepoint x Affiliative Response | 0.02 | 1, 56 | .88 | .00 | 0.00 | (1, 54) | .97 | .00 |
|  | Timepoint x Social Sensitivity | 0.22 | 1, 59 | .64 | .00 | 0.01 | (1, 57) | .91 | .00 |
|  | Affiliative Response x Social Sensitivity | 0.66 | 1, 80 | .42 | .01 | 0.08 | (1, 77) | .78 | .00 |
|  | Timepoint x Affective Response x Social Sensitivity | 0.36 | 1, 56 | .55 | .01 | 0.15 | (1, 54) | .70 | .00 |
| **d. Social Media Interactions** | | | | | | | | | |
| **Affective Response**^e^ | |  |  |  |  |  |  |  |  |
|  | Timepoint^f^ | 1.41 | 1,22 | .25 | .06 | 0.02 | (1,28) | .89 | .00 |
|  | Affective Response | 0.67 | 1,44 | .42 | .02 | 0.18 | (1,47) | .67 | .00 |
|  | Social Sensitivity^g^ | 13.57 | 1,40 | <.001 | .25 | 15.44 | (1,41) | <.001 | .27 |
|  | Timepoint x Affective Response | 1.33 | 1,22 | .26 | .06 | 1.26 | (1,29) | .27 | .04 |
|  | Timepoint x Social Sensitivity | 0.43 | 1,21 | .52 | .02 | 0.06 | (1,27) | .82 | .00 |
|  | Affective Response x Social Sensitivity | 0.11 | 1,41 | .74 | .00 | 2.23 | (1,42) | .14 | .05 |
|  | Timepoint v Affective Response x Social Sensitivity | 5.83 | 1,21 | .025 | .21 | 4.34 | (1,27) | .05 | .14 |
| **Affiliative Response**^e^ | |  |  |  |  |  |  |  |  |
|  | Timepoint^f^ | 1.50 | 1,22 | .23 | .06 | 0.04 | (1,28) | .84 | .00 |
|  | Affiliative Response | 1.10 | 1,42 | .30 | .03 | 0.20 | (1,43) | .66 | .00 |
|  | Social Sensitivity^g^ | 13.23 | 1,41 | <.001 | .25 | 13.00 | (1,41) | <.001 | .24 |
|  | Timepoint x Affiliative Response | 0.38 | 1,22 | .55 | .02 | 0.60 | (1,27) | .45 | .02 |
|  | Timepoint x Social Sensitivity | 0.97 | 1,22 | .34 | .04 | 0.35 | (1,26) | .56 | .01 |
|  | Affiliative Response x Social Sensitivity | 0.10 | 1,39 | .75 | .00 | 2.45 | (1,37) | .13 | .06 |
|  | Timepoint x Affective Response x Social Sensitivity | 7.47 | 1,21 | .01 | .26 | 5.26 | (1,25) | .03 | .17 |
| **e. Texting Interactions** | | | | | | | | | |
| **Affective Response**^e^ | |  |  |  |  |  |  |  |  |
|  | Timepoint^f^ | 1.32 | 1, 62 | .26 | .02 | 0.00 | (1, 66) | .95 | .00 |
|  | Affective Response | 0.77 | 1, 109 | .38 | .01 | 0.39 | (1, 112) | .53 | .00 |
|  | Social Sensitivity^g^ | 22.53 | 1, 100 | < .001 | .18 | 21.84 | (1, 103) | < .001 | .18 |
|  | Timepoint x Affective Response | 0.00 | 1, 67 | .97 | .00 | 0.43 | (1, 70) | .57 | .01 |
|  | Timepoint x Social Sensitivity | 0.35 | 1, 63 | .56 | .01 | 0.19 | (1, 67) | .66 | .00 |
|  | Affective Response x Social Sensitivity | 2.37 | 1, 91 | .13 | .03 | 0.17 | (1, 94) | .68 | .00 |
|  | Timepoint v Affective Response x Social Sensitivity | 1.21 | 1, 60 | .28 | .02 | 0.14 | (1, 64) | .71 | .00 |
| **Affiliative Response**^e^ | |  |  |  |  |  |  |  |  |
|  | Timepoint^f^ | 0.56 | 1, 63 | .46 | .01 | 0.20 | (1, 66) | .66 | .00 |
|  | Affiliative Response | 0.13 | 1, 96 | .72 | .00 | 3.81 | (1, 98) | .05 | .04 |
|  | Social Sensitivity^g^ | 21.23 | 1, 101 | < .001 | .17 | 18.50 | (1, 103) | < .001 | .15 |
|  | Timepoint x Affiliative Response | 0.29 | 1, 62 | .59 | .00 | 1.26 | (1, 65) | .27 | .02 |
|  | Timepoint x Social Sensitivity | 0.37 | 1, 64 | .54 | .01 | 0.89 | (1, 67) | .35 | .01 |
|  | Affiliative Response x Social Sensitivity | 0.09 | 1, 88 | .76 | .00 | 0.10 | (1, 90) | .75 | .00 |
|  | Timepoint x Affective Response x Social Sensitivity | 0.13 | 1, 59 | .72 | .00 | 0.94 | (1, 62) | .34 | .02 |
| g. **Online Social Interactions** | | | | | | | | | |
| **Affective Response**^e^ | |  |  |  |  |  |  |  |  |
|  | Timepoint^f^ | 1.20 | 1, 72 | .28 | .02 | 0.01 | (1, 78) | .91 | .00 |
|  | Affective Response | 0.24 | 1, 121 | .62 | .00 | 0.80 | (1, 122) | .37 | .01 |
|  | Social Sensitivity^g^ | 22.90 | 1, 116 | < .001 | .17 | 24.43 | (1, 117) | < .001 | .17 |
|  | Timepoint x Affective Response | 0.09 | 1, 73 | .77 | .00 | 0.00 | (1, 79) | >.99 | .00 |
|  | Timepoint x Social Sensitivity | 0.42 | 1, 71 | .52 | .01 | 0.26 | (1, 77) | .61 | .00 |
|  | Affective Response x Social Sensitivity | 2.08 | 1, 103 | .15 | .02 | 0.00 | (1, 104) | .96 | .00 |
|  | Timepoint v Affective Response x Social Sensitivity | 1.69 | 1, 67 | .20 | .02 | 0.73 | (1, 72) | .40 | .01 |
| **Affiliative Response**^e^ | |  |  |  |  |  |  |  |  |
|  | Timepoint^f^ | 0.55 | 1, 74 | .46 | .01 | 0.07 | (1, 78) | .79 | .00 |
|  | Affiliative Response | 0.36 | 1, 113 | .55 | .00 | 1.75 | (1, 114) | .19 | .02 |
|  | Social Sensitivity^g^ | 22.43 | 1, 118 | < .001 | .16 | 21.07 | (1, 119) | < .001 | .15 |
|  | Timepoint x Affiliative Response | 0.12 | 1, 72 | .73 | .00 | 1.67 | (1, 76) | .20 | .02 |
|  | Timepoint x Social Sensitivity | 0.29 | 1, 74 | .59 | .00 | 0.51 | (1, 78) | .48 | .01 |
|  | Affiliative Response x Social Sensitivity | 0.04 | 1, 105 | .84 | .00 | 0.04 | (1, 106) | .84 | .00 |
|  | Timepoint x Affective Response x Social Sensitivity | 0.02 | 1, 69 | .89 | .00 | 0.07 | (1, 72) | .79 | .00 |

^a^Anxiety=total score on the 7-item Generalized Anxiety Disorder Scale (GAD-7)^1^; Depression=total score on the 8-item Patient Health Questionnaire (PHQ-8)^2^.

^b^ Alpha threshold set to .025 applying a Bonferroni correction for the two outcomes of interest (anxiety and depression symptoms).

^c^η ^2^_p_ is partial eta-squared, where .01, .06 and .14 are small, medium, and large effect sizes, respectively^4^.

^d^All interactions includes all in-person, phone, social media and texting interactions.

^e^At each EMA participants reported their affective and affiliative response to their most important social interaction in the past two hours on a scale ranging from 0=*Very Unhappy* to 100=*Very Happy* and from 0=*Left out* to 100=*Included*, respectively.

^f^ Timepoint*=*baseline vs. 1-month follow-up.

^g^Social Sensitivity= total score on the Online and Offline Social Sensitivity Questionnaire (O^2^S^3^)^3^.

**References**

1. Spitzer RL, Kroenke K, Williams JBW, Löwe B. A brief measure for assessing generalized anxiety disorder: the GAD-7. Arch Intern Med. May 22, 2006;166(10):1092-1097. [doi: 10.1001/archinte.166.10.1092] [Medline: 16717171]
2. Kroenke K, Strine TW, Spitzer RL, Williams JBW, Berry JT, Mokdad AH. The PHQ-8 as a measure of current depression in the general population. J Affect Disord. Apr 2009;114(1-3):163-173. [doi: 10.1016/j.jad.2008.06.026]
3. Andrews JL, Khin AC, Crayn T, Humphreys K, Schweizer S. Measuring online and offline social rejection sensitivity in the digital age. Psychol Assess. Aug 2022;34(8):742-751. [doi: 10.1037/pas0001136] [Medline: 35482615]
4. Richardson JTE. Eta squared and partial eta squared as measures of effect size in educational research. Educ Res Rev. Jan 2011;6(2):135-147. [doi: 10.1016/j.edurev.2010.12.001]
