# Supplementary material for: Momentary Mood and Affiliation Following Social Interactions in the Digital Age: Longitudinal Study Investigating Associations With Anxiety and Depression
Source: JMIR Ment Health. 2026 Aug 10;13:e94753. doi: 10.2196/94753 (PMC13455724; doi:10.2196/94753)
Supplement: Supplementary file 2 [file mental-v13-e94753-s002.docx]

Multimedia Appendix 2: Survey questionnaires

The following self-report questionnaires were completed online by all participants, following informed consent, at baseline (including demographic questions) and 1-month follow-up (excluding demographic questions).

Attention checks were included after every two questionnaires (three in total).

Demographic questions

- Age
  - please enter your age in years ________________
- Please indicate what country you live in (we need this information to send notifications at times you can respond to them in Part 2. If you prefer not to say, you will be re-directed out of this study)

a. Australia
b. UK
c. USA
d. Ireland
e. Canada
f. South Africa
g. NZ
h. Prefer not to say

IF AUSTRALIA, THE FOLLOWING WILL APPEAR:

- Which Australian time zone are you in (we need this information to send notifications at times you can respond to them in Part 2)?
  - Eastern Australia (GMT +10:00 - Syd / Melb / Bris)
  - Central Australia (GMT +9:30 - Adelaide, Darwin)
  - Western Australia (GMT +8:00 - Perth)

IF USA OR CANADA, THE FOLLOWING WILL APPEAR:

- Which US / Canadian time zone are you in (we need this information to send notifications at times you can respond to them in Part 2)?
  - Hawaii (GMT -10:00)
  - Alaska (GMT -9:00)
  - Pacific Time (GMT -8:00 – L.A., Vancouver)
  - Mountain Time (GMT – 7:00 – Denver, Edmonton)
  - Central Time (GMT – 6:00 Chicago, Winnipeg)
  - Eastern Time (GMT – 5:00 – New York, Toronto)
  - Atlantic Time (GMT – 4:00 – Halifax)
  - Newfoundland (GMT -3:30)
- Please select which gender you identify with:

a. Female
b. Male
c. Non-binary
d. I use a different term (free text response)
e. Prefer not to say

- Please select which ethnicity you identify with:

a. Aboriginal or Torres Strait Islander
b. Black
c. Asian
d. White
e. Hispanic
f. Mixed
g. Other
h. Prefer not to say

- What is your current/ highest level of education?

a. Primary School
b. High school
c. Professional/vocational training
d. University
e. Prefer not to say

- How well off do you think you are? By well off we mean how much money/wealth you have.

a. Not at all
b. Not very
c. Fairly
d. Rather
e. Very
f. Prefer not to say

- Have you ever been diagnosed with a mental health condition?
  - Yes
  - No
  - Prefer not to say

IF YES THE FOLLOWING WILL APPEAR:

- Please select all that apply to you:

a. Depression
b. Anxiety disorder (any)
c. Eating disorder
d. Bipolar disorder (manic depression)
e. Schizophrenia
f. Post-traumatic stress disorder
g. Obsessive-compulsive disorder
h. Other (please specify)
i. Prefer not to say

- Please indicate whether you are currently suffering from (please tick all that apply):

a. Depression
b. Anxiety disorder (any)
c. Eating disorder
d. Bipolar disorder (manic depression)
e. Schizophrenia
f. Post-traumatic stress disorder
g. Obsessive-compulsive disorder
h. Other (please specify)
i. Prefer not to say

- Do you have a history of a neurodevelopmental or neurological disorder?
  - Yes
  - No
  - Prefer not to say

IF YES THE FOLLOWING WILL APPEAR:

- Please select all that apply to you:

a. Epilepsy
b. Seizures
c. Attention deficit/Hyperactivity disorder
d. Autism Spectrum Disorder
e. Asperger Syndrome
f. Reading disorder (dyslexia)
g. Writing disorder (dysgraphia)
h. Calculation disorder (dyscalculia)
i. Other (please specify)
j. Prefer not to say

**Generalized Anxiety Disorder 7 Scale (GAD-7)**^1^

How often have you been bothered by each of the following problems during the past **two weeks**? For each problem, put an “X” in the box beneath the answer that best describes how you have been feeling.

|  | Not at all | Several days | More than half the days | Nearly every day |
| --- | --- | --- | --- | --- |
| 1. Feeling nervous, anxious or on edge |  |  |  |  |
| 2. Not being able to stop or control worrying |  |  |  |  |
| 3. Worrying too much about different things |  |  |  |  |
| 4. Trouble relaxing |  |  |  |  |
| 5. Being so restless it is hard to sit still |  |  |  |  |
| 6. Becoming easily annoyed or irritable |  |  |  |  |
| 7. Feeling afraid as if something awful might happen |  |  |  |  |

**Patient Health Questionnaire Adolescent (PHQ-8)**^2^

How often have you been bothered by each of the following symptoms during the past **two weeks**? For each symptom, put an “X” in the box beneath the answer that best describes how you have been feeling.

|  | Not at all | Several days | More than half the days | Nearly every day |
| --- | --- | --- | --- | --- |
| 1. Little interest in pleasure or doing things |  |  |  |  |
| 2. Feeling down, depressed, irritable or hopeless |  |  |  |  |
| 3. Trouble falling asleep, staying asleep or sleeping too much |  |  |  |  |
| 4. Feeling tired or having little energy |  |  |  |  |
| 5. Poor appetite, weight loss or overeating |  |  |  |  |
| 6. Feeling bad about yourself – or that you are a failure or have let yourself or your family down |  |  |  |  |
| 7. Trouble concentrating on things, such as school work, reading or watching television |  |  |  |  |
| 8. Moving or speaking so slowly that other people could have noticed? Or the opposite – being so fidgety or restless that you have been moving around a lot more than usual |  |  |  |  |

**Social Sensitivity Scale**^3^

Below we listed a number of statements that describe how you may feel in social situations.

For each statement, please indicate how much you agree with it on a scale from 0-3.

| **Strongly Disagree** | **Moderately Disagree** | **Moderately Agree** | **Strongly Agree** |
| --- | --- | --- | --- |
| 0 | 1 | 2 | 3 |

Please respond to each item how much you ***generally* agree with it / how well it generally represents you**. There are no right or wrong answers.

|  | **Statement** | 1 | 2 | 3 | 4 |
| --- | --- | --- | --- | --- | --- |
| 1. | I worry about the effect I have on other people | 1 | 2 | 3 | 4 |
| 2. | I delete my social media post if I don’t get the responses I wanted | 1 | 2 | 3 | 4 |
| 3. | I feel that people generally like me | 1 | 2 | 3 | 4 |
| 4. | I feel anxious before posting anything on social media | 1 | 2 | 3 | 4 |
| 5. | I always expect criticism | 1 | 2 | 3 | 4 |
| 6. | I care about what people feel about me | 1 | 2 | 3 | 4 |
| 7. | Initiating contact online with others, such as sending friend requests, is not something I feel comfortable doing | 1 | 2 | 3 | 4 |
| 8. | When I update my social media status and no one comments on it, I tend to be disappointed | 1 | 2 | 3 | 4 |
| 9. | I check responses to my posts on social media very often | 1 | 2 | 3 | 4 |
| 10. | If others knew the real me, they would not like me | 1 | 2 | 3 | 4 |
| 11. | My value as a person depends enormously on what others think of me | 1 | 2 | 3 | 4 |
| 12. | When I update my social media status, I expect others to comment on it | 1 | 2 | 3 | 4 |
| 13. | I worry about being criticised for things I have said or done | 1 | 2 | 3 | 4 |
| 14. | I feel others do not understand me | 1 | 2 | 3 | 4 |
| 15. | I prefer to post on social media sites where I can stay anonymous (i.e. without revealing my name or identity) | 1 | 2 | 3 | 4 |
| 16. | If other people knew what I am really like, they would think less of me | 1 | 2 | 3 | 4 |
| 17. | I worry about what others think of me | 1 | 2 | 3 | 4 |
| 18. | I feel insecure if I don’t receive positive responses to my social media posts | 1 | 2 | 3 | 4 |

**EMA Questions** (Bespoke questions, informed by an academic literary review^4–8^)

1. How do you feel right now?
   - *Sliding scale* *from “Very unhappy” to “Very happy”*
2. Are you trying to change the way you feel right now?
   - *Multiple choice (multiple options)*
     - Yes, by distracting myself
     - Yes, by dealing with the problem
     - Yes, by doing something fun
     - Yes, by thinking about the problem differently
     - Yes, by avoiding the problem
     - Yes, by spending time with friends/family
     - Yes, by accepting the situation
     - Other
     - No
3. What are you doing right now?  (Multiple choice)
   - *Multiple choice (multiple options)*
     - Working/studying/schoolwork
     - Commuting (to/from work/school)
     - Resting
     - Social media
     - Being on the internet
     - Leisure
     - Chores
     - Childcare
     - Waiting/Queuing in line
     - Watching TV
     - Other
4. Right now, are you?
   - *Multiple choice (single option)*
     - Alone
     - With a friend(s) / family
     - With someone(s) you don’t know / a stranger(s)
5. In the last two hours, have you worried about being judged or evaluated by others?
   - *Sliding scale “Not at all” to “Very much”*
     1. In-person
     2. Online

6. Have you connected socially in-person (in real-life) or online (social media or other technology including reading or posting on social media) in the last two hours?
   - *Multiple choice (single option)*
     - Yes
     - No

If Yes:

7. How many social connections have you had in the last two hours?
   - *Sliding scale from “1” to “20+”*
     - *In-person*
     - Digital / online (e.g. writing messages, sharing, ‘liking’ or commenting on a post, chatting in-game, etc)
8. In the last two hours, how much *time (in minutes) did you spend passively* reading or viewing posts or messages from …
   - *Slider “1 – 120 minutes”*
     - People you know
     - Strangers (e.g., influencers)
9. Now, please think about the single most important connection you had in the past two hours, was it ….
   - *Multiple Choice (single choice)*
     - In-person / real-life
     - Phone / voice / video call
     - Text message / instant message / SMS / WhatsApp / Email
     - Social media (e.g. Instagram, TikTok, Facebook, Snapchat, LinkedIn)
     - Other (please specify)
10. For this same, most important connection, what *type of connection* was it (how many people interacted)?
    - *Multiple Choice (single choice)*
      - One-way (e.g. alone posting / viewing posts, messaged with no response)
      - Two-way (i.e., me and another person interacting)
      - Private Group (i.e. multiple people but not public)
      - Public (i.e., anyone could join)
11. For this same, most important connection, *who was* involved (whose posts did you view / who did you connect or interact with)?
    - *Multiple Choice (multiple options)*
      - Spouse / Partner
      - Friend(s)
      - Family
      - Acquaintance / Colleague / Classmate / Neighbour
      - Boss / Teacher
      - Someone I don’t know / Stranger
12. For this same social connection, *who initiated* contact?
    - *Multiple choice (single option)*
      - *Me*
      - Someone else
13. For this same social connection, what best describes your *reason* for the interaction?
    - *Multiple choice (multiple options)*
      - I wanted to ….
        - Arrange / cancel meeting up
        - Support a friend
        - Share problems / bad news
        - Share good news / cool stuff
        - Make friends / new contacts
        - Browse other’s social media feeds (for entertainment)
        - Browse other’s feeds to compare myself/my life
        - Comment on other’s social media feeds
        - Get info / news
        - Other (please specify)
14. For this same social connection, how did the social connection make you feel overall?
    - *Sliding scale* *from*
      - *“Very Unhappy” to “Very Happy”*
      - *“Left out”* to *“Included”*
15. For this same social connection, how did the social connection make you feel specifically?
    - *Multiple choice (multiple options)*
      - I felt ….
        - Content
        - Excited
        - Proud
        - Confident
        - Reassured things are / would be OK
        - Competent
        - Disappointed
        - Worried about being judged negatively
        - Insecure
        - Angry
        - Sad
        - Stressed
        - Lonely
        - Other (please specify)

If No:

11. Is it typical for you to have no social connection for two hours at this time of day?
    - *Multiple Choice (single choice)*
      1. No
      2. Yes, but only on this day of the week
      3. Yes, most days
12. What best describes why you have had no social connection in the last two hours?
    - *Multiple Choice (single choice)*
      1. Happy being alone
      2. I need a break from social interactions
      3. I’m worried about being judged
      4. No one’s available
      5. I feel an outsider / left-out
      6. Busy / working
      7. Other (please specify):

**If “Happy being alone”**

9. Do you usually enjoy social interactions?
   - *Multiple Choice (single choice)*
     1. Yes
     2. No
10. Was there a particular incident that meant you needed a break from social interactions?
    - *Multiple Choice (single choice)*
      1. Yes
      2. No
11. If yes, what sort of incident was it?
    - *Multiple Choice (single choice)*
      1. Someone was mean or judgemental
      2. I had an argument
      3. It’s exhausting to interact with others
      4. Other (please specify)

11. If no, Why were you happy being alone in the last two hours?

- *Multiple Choice (multiple options)*
  1. It’s easier to relax
  2. I like silence
  3. I can be myself
  4. I have my best ideas alone
  5. It’s my “me time”
  6. Other (please specify)

**If “I need a break from social interaction”**

9. Do you usually enjoy social interactions?
   - *Multiple Choice (single choice)*
     1. Yes
     2. No
10. Was there a particular incident that meant you needed a break from social interactions?
    - *Multiple Choice (single choice)*
      1. Yes
      2. No
11. If yes, what sort of incident was it?
    - *Multiple Choice (single choice)*
      1. Someone was mean or judgemental
      2. I had an argument
      3. It’s exhausting to interact with others right now
      4. Other (please specify)

11. If no, Why did you need a break from social interaction?

- *Open text box*

**If “I’m worried about being judged”**

9. Was there a particular incident that caused you to worry about being judged?
   - *Multiple Choice (single choice)*
     1. Yes
     2. No
10. If yes, What sort of incident was it?
    - *Multiple Choice (single choice)*
      1. Someone expressed judgement about me in-person
      2. Someone expressed judgement about me online
      3. I felt like someone was judging me in-person
      4. I felt like someone was judging me online
      5. Someone expressed judgement about someone else to me in-person
      6. Someone expressed judgement about someone else online
      7. Other (please specify)

10.If no, Is there another reason you were worried about being judged in the last two hours?

- *Open text box*

**If “No one’s available”**

9. How did you know no-one was available?
   - *Multiple Choice (single choice)*
     1. I knew everyone was busy because of their socials
     2. I know everyone was busy because I know what all my friends and family do at this time
     3. I just assumed everyone would be at work / school
     4. I just assumed everyone would be busy with something else
     5. Other (please specify)
10. Would you have liked to have connected socially in the last two hours?
    - *Multiple Choice (single choice)*
      1. Yes – in-person
      2. Yes - online
      3. No
11. Did you think of spending time browsing social media?
    - *Multiple Choice (single choice)*
      1. No – I didn’t want to socialise
      2. No - I don’t feel like this is social connection if it is one-way
      3. Yes - but I didn’t because (please specify reason)

**If “I feel an outsider / left-out”:**

9. Was there a particular incident that caused you to feel an outsider / left-out?
   - *Multiple Choice (single choice)*
     1. Yes
     2. No
10. If yes, What sort of incident was it?
    - *Multiple Choice (single choice)*
      1. Someone was mean or judgemental about me in-person
      2. Someone was mean or judgemental about me online
      3. I wasn’t invited to an in-person event
      4. I wasn’t invited to an online event
      5. I was left out of a new group online (e.g. WhatsApp chat)
      6. I don’t use social media and my connections all do
      7. Other (please specify)
11. Would you have liked to have connected socially in the last two hours?
    - *Multiple Choice (single choice)*
      1. Yes – in-person
      2. Yes - online
      3. No
12. If yes, Did you think of spending time browsing social media?
    - *Multiple Choice (single choice)*
      1. No – I didn’t want to socialise
      2. No - I don’t feel like this is social connection if it is one-way
      3. Yes - but I didn’t because (please specify reason)

**If “Busy / working”:**

10. Would you have liked to have connected socially in the last two hours?
    - *Multiple Choice (single choice)*
      1. Yes – in-person
      2. Yes - online
      3. No
11. Have you been in a situation where you would have connected socially if less busy?
    - *Multiple Choice (multiple options)*
      1. Yes – I am in an office / workspace with other people
      2. Yes – I have run errands with other people around
      3. Yes – I would have connected socially online
      4. Yes – other (please specify)
      5. No – I wouldn’t have wanted to anyway
      6. No – I haven’t seen anyone or had internet access
12. Do you consider conversations or connections you make with people in the course of your work / busy day social connections?
    - *Multiple Choice (single choice)*
      1. Yes – I try to connect with anyone I come into contact with
      2. Maybe – I guess anytime you talk to someone it is a social connection
      3. No – I don’t talk to people I don’t know really well / at work

# References

1.	Spitzer RL, Kroenke K, Williams JBW, Löwe B. A brief measure for assessing generalized anxiety disorder: the GAD-7. *Arch Intern Med*. 2006;166(10):1092-1097. doi:10.1001/archinte.166.10.1092

2.	Kroenke K, Strine TW, Spitzer RL, Williams JBW, Berry JT, Mokdad AH. The PHQ-8 as a measure of current depression in the general population. *J Affect Disord*. 2009;114(1-3):163-173. doi:10.1016/j.jad.2008.06.026

3.	Andrews JL, Khin AC, Crayn T, Humphreys K, Schweizer S. Measuring online and offline social rejection sensitivity in the digital age. *Psychol Assess*. 2022;34(8):742-751. doi:10.1037/pas0001136

4.	Lyubomirsky S, King L, Diener E. The benefits of frequent positive affect: does happiness lead to success? *Psychol Bull*. 2005;131(6):803-855. doi:10.1037/0033-2909.131.6.803

5.	Subrahmanyam K, Frison E, Michikyan M. The relation between face‐to‐face and digital interactions and self‐esteem: a daily diary study. *Human Behav Emerg Technol*. 2020;2(2):116-127. doi:10.1002/hbe2.187

6.	Garrett SL, Burnell K, Armstrong‐Carter EL, Prinstein MJ, Telzer EH. Linking video chatting, phone calling, text messaging, and social media with peers to adolescent connectedness. *J Res Adolesc*. 2023;33(4):1222-1234. doi:10.1111/jora.12871

7.	Masciantonio A, Bourguignon D. Motivation scale for using social network sites: comparative study between Facebook, Instagram, Twitter, Snapchat and LinkedIn. *Psychol Belg*. 2023;63(1):30-43. doi:10.5334/pb.1161

8.	Tomova L, Andrews JL, Blakemore SJ. The importance of belonging and the avoidance of social risk taking in adolescence. *Dev Rev*. 2021;61:100981. doi:10.1016/j.dr.2021.100981
